# New Approach Methodologies for the Endocrine Activity Toolbox: Environmental Assessment for Fish and Amphibians

**DOI:** 10.1002/etc.5584

**Published:** 2023-03-20

**Authors:** Constance A. Mitchell, Natalie Burden, Mark Bonnell, Markus Hecker, Thomas H. Hutchinson, Magdalena Jagla, Carlie A. LaLone, Laurent Lagadic, Scott G. Lynn, Bryon Shore, You Song, Sara M. Vliet, James R. Wheeler, Michelle R. Embry

**Affiliations:** a The Health and Environmental Sciences Institute, Washington, DC, USA; b National Centre for the 3Rs (NC3Rs), London, United Kingdom; c Environment and Climate Change Canada, Ottawa, Canada; d Toxicology Centre and School of the Environment & Sustainability, University of Saskatchewan, Saskatoon, Canada; e Reckitt R&D, Hull, United Kingdom; f Office of Research and Development, Great Lakes Toxicology & Ecology Division, US Environmental Protection Agency, Duluth, Minnesota; g Research and Development, Crop Science, Environmental Safety, Bayer, Monheim am Rhein, Germany; h Office of Pesticide Programs, US Environmental Protection Agency, Washington, DC; i Norwegian Institute for Water Research, Oslo, Norway; j Office of Research and Development, Scientific Computing and Data Curation Division, US Environmental Protection Agency, Duluth, Minnesota; k Corteva Agriscience, Bergen op Zoom, The Netherlands

**Keywords:** Adverse outcome pathway, Endocrine-disrupting compounds, In vitro toxicology

## Abstract

Multiple in vivo test guidelines focusing on the estrogen, androgen, thyroid, and steroidogenesis pathways have been developed and validated for mammals, amphibians, or fish. However, these tests are resource-intensive and often use a large number of laboratory animals. Developing alternatives for in vivo tests is consistent with the replacement, reduction, and refinement principles for animal welfare considerations, which are supported by increasing mandates to move toward an “animal-free” testing paradigm worldwide. New approach methodologies (NAMs) hold great promise to identify molecular, cellular, and tissue changes that can be used to predict effects reliably and more efficiently at the individual level (and potentially on populations) while reducing the number of animals used in (eco)toxicological testing for endocrine disruption. In a collaborative effort, experts from government, academia, and industry met in 2020 to discuss the current challenges of testing for endocrine activity assessment for fish and amphibians. Continuing this cross-sector initiative, our review focuses on the current state of the science regarding the use of NAMs to identify chemical-induced endocrine effects. The present study highlights the challenges of using NAMs for safety assessment and what work is needed to reduce their uncertainties and increase their acceptance in regulatory processes. We have reviewed the current NAMs available for endocrine activity assessment including in silico, in vitro, and eleutheroembryo models. New approach methodologies can be integrated as part of a weight-of-evidence approach for hazard or risk assessment using the adverse outcome pathway framework. The development and utilization of NAMs not only allows for replacement, reduction, and refinement of animal testing but can also provide robust and fit-for-purpose methods to identify chemicals acting via endocrine mechanisms.

## INTRODUCTION

Endocrine pathways regulate growth, development, reproduction, metabolism, and tissue function in vertebrates (US Environmental Protection Agency [USEPA], 2022b). Examples of endocrine pathways important for the functions listed are estrogen, androgen, thyroid, and steroidogenesis modalities, which are the focus of regulatory programs such as the USEPA’s Office of Pesticide Programs (OPP). A chemical that alters the function of an endocrine system and causes subsequent adverse effects in an intact organism, its progeny, or (sub)populations is referred to as an endocrine-disrupting chemical (EDC; International Programme on Chemical Safety, 2002). Endocrine activity, by contrast, describes an interaction with the endocrine system observed during a toxicological study that may or may not lead to an adverse effect. There is also scientific consensus that the World Health Organization (WHO)/International Programme on Chemical Safety definition does not necessarily mean that the adverse effect has to be demonstrated in an intact test animal but may be shown in adequately validated alternative test systems predictive of adverse effects in humans and/or wildlife (Solecki et al., 2017). Endocrine-disrupting chemicals may alter the endocrine system by mimicking endogenous hormones (e.g., binding to receptors) or altering the synthesis, metabolism, and transport of hormones (Schneider et al., 2019) and are of concern for both human and ecological health. Testing requirements related to identification of EDCs are developing worldwide but vary in approaches. These range from test requirements for all substances being registered or marketed for a particular use to exposure- and production volume–based scenarios to specific testing triggered by emerging concerns (Table 1).

Initial testing for endocrine activity typically involves short-term in vivo and in vitro assays used to evaluate the potential for interactions of a chemical with select endocrine pathways. Where activity is identified to be of concern, higher-tier in vivo testing is subsequently required to confirm the activity and establish adversity. Multiple in vivo test guidelines have been validated for mammals, amphibians, and fish by the Organisation for Economic Co-operation and Development (OECD) and the USEPA (OPPTS 890 test guidelines; Burden et al., 2022; OECD, 2021a). These guidelines often require the use of a large number of laboratory animals (Russell & Burch, 1959). Because of these animal welfare and economic concerns, there are mandates to move toward an “animal-free” testing paradigm worldwide (Frank R. Lautenberg Chemical Safety for the 21st Century Act, 2016; Oziel, 2017; Wheeler, 2019).

### The potential for expanding the use of new approach methodologies (NAMs) in endocrine activity testing and assessment

New approach methodologies refer to “any technology, methodology, approach, or combination thereof that can be used to provide information on chemical hazard and risk assessment that avoids the use of [intact] animals” (OECD, 2005). However, the term NAM can also include whole-organism assays using some invertebrates or the nonprotected eleutheroembryonic life stages of vertebrates (e.g., fish and amphibians, typically prior to independent feeding; Embry et al., 2010; von Hellfeld et al., 2020). For the purposes of the present study, we refer to NAMs as all (eco)toxicological tools other than traditional in vivo toxicity tests. The USEPA (2021) similarly defines NAMs as “any technology, methodology, approach, or combination that can provide information on chemical hazard and risk assessment to avoid the use of animal testing.” Generally, NAMs can provide information on molecular changes, molecular/cellular perturbations, toxicokinetics, toxicodynamics, and toxicity mechanisms rather than measuring apical endpoints that are assessed in vivo. New approach methodologies hold great promise to increase the efficiency of screening testing to inform regulatory decisions, while having the added benefit of reducing the need for more resource-intensive studies which are reliant on large numbers of animals (OECD, 2005). Currently, however, there is limited confidence in using these tools broadly for hazard and risk assessment (Burden et al., 2022); and to date NAMs have been primarily used for chemical screening, classification, or prioritization or to assist in providing a mechanistic understanding of effects observed in in vivo studies. This is partly because many NAMs lack the complex biological processes that in vivo systems possess, such as absorption, distribution, metabolism, and excretion (ADME) processes or cross talk among multiple tissues or organs. Furthermore, insufficient mechanistic knowledge of the biochemical pathways of interest may pose issues concerning the use and interpretation of NAM data. This collaborative effort strives to summarize the current state of the science regarding the use of NAMs to identify and predict chemical-induced endocrine effects and aims to identify steps needed to increase acceptance of NAMs in regulatory processes.

### Adverse outcome pathways as a framework to support NAM development and application

**FIGURE 1:**
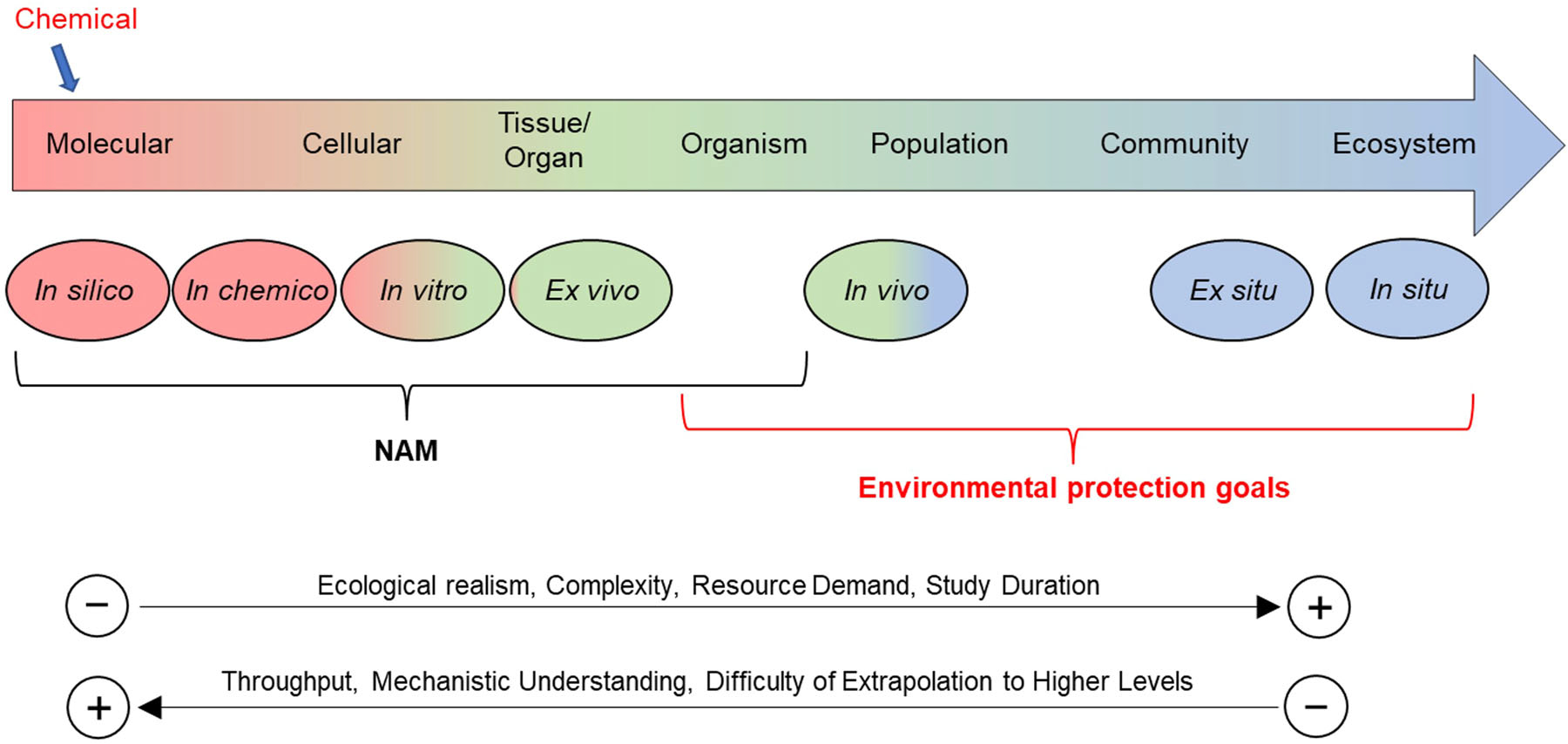
Current overview: levels of biological organization and tools in support of chemical ecological assessment. NAM = new approach methodology.

The adverse outcome pathway (AOP) framework has been developed to support a more mechanistically focused approach to toxicity testing and drive the development and integration of toxicodynamic NAMs in safety assessment. Adverse outcome pathways are constructed using available information to define the sequence of molecular and cellular events required to produce an adverse effect on individuals, with potential consequences at the population level when organisms are exposed to a substance. Adverse outcome pathways entail linking a molecular initiating event, which describes the initial interaction of a chemical with a biomolecule, through a cascade of downstream key events (KEs) occurring at multiple levels of biological organization (i.e., cellular, tissue, organ), to an adverse outcome of regulatory concern (Ankley et al., 2010). The strength of the causal relationships between KEs (termed KE relationships) can be evaluated by weight-of-evidence (WoE) considerations (Collier et al., 2016), integrating evidence streams along the AOP. Changes to an organism induced by perturbations of the pathway can then be mapped along the AOP. With sufficient understanding and development of quantitative endocrine AOPs, NAMs can be developed and used in place of animal toxicity tests to determine whether a substance induces early KEs within the AOP and ultimately help to predict if an adverse outcome is likely. The estrogen, androgen, thyroid, and steroidogenesis modalities are of interest in the current regulatory paradigms. These pathways tend to be conserved across species, are relatively well studied, and in some cases have a robust set of in vivo adverse outcome data. Figure 1 maps the different methods and approach types that are available to support general chemical assessment for ecological endpoints along an AOP. Such organizational thinking can be applied for chemicals acting on endocrine pathways. However, the extrapolation from individual-based adverse outcomes to a population-relevant apical effect, in line with the protection goals of most environmental assessments, remains a significant challenge (Crane et al., 2019; Lagadic et al., 2020; Perkins et al., 2019).

It is worth noting that while NAMs hold promise for high-throughput screening and assessment with the added benefit of reducing dependence on traditional animal tests, they are not necessarily a one-to-one replacement. However, their data should be considered a key component in an integrated WoE approach for decision-making. A WoE approach uses a combination of information from several sources, with weight given to the available evidence based on expert judgment or reliability factors (e.g., quality of the data, consistency of results, and relevance of the information; European Chemicals Agency, 2016). Despite their limitations, NAMs provide the possibility to incorporate mechanistic information into the chemical assessment process, to increase the efficiency of such assessments, and to ultimately reduce the overall numbers of animals used in ecotoxicology testing and assessment. In particular, in silico, in vitro, and eleutheroembryo models are more rapid and higher-throughput than traditional in vivo approaches, allowing more chemicals to be screened with fewer resources and in less time.

### Aims of our review

In a collaborative effort led by the Health and Environmental Sciences Institute (HESI) and the United Kingdom’s National Centre for the 3Rs, experts from government, academia, and industry met in early 2020 to discuss the current challenges of endocrine testing for fish and amphibians (Burden et al., 2022). In continuing the cross-sector initiative, the present study outlines the current state of the science for evaluating the endocrine-disrupting potential of chemicals in amphibians and fish using NAMs across the estrogen, androgen, thyroid, and steroidogenesis pathways. In the present study, we discuss the use of NAMs, their current and potential roles for ecotoxicology testing, and their potential to be used in paradigms like AOPs and integrated approaches to testing and assessments (IATAs). We also discuss the challenges of using NAMs for endocrine assessments and what is needed to reduce the uncertainties with such approaches. Movement toward the development, validation, and utilization of NAMs will lead to the replacement, reduction, and refinement of animal use needed for endocrine activity assessments while increasing the robustness and fitness for purpose of regulatory EDC assessments.

## USING PROBLEM FORMULATION TO ENSURE THAT NAMs ARE FIT FOR PURPOSE

Problem formulation is defined as a systematic approach that identifies the factors critical to a specific assessment and considers the purpose, scope, and depth of the necessary analysis, analytical approach, available resources and outcomes, and overall management goal (Solomon et al., 2016). Because management goals may vary by sector, legislation, and geographic region, the specific regulatory context must be taken into account. One example of this is the difference between approaches to the regulation of EDCs in Canada, the United States, and Japan, where a risk-based approach is used, and in the European Union, where a hazard-based approach is employed (i.e., a lack of consideration of exposure in the assessment; Burden et al., 2022). Therefore, data and assessment methodology will vary significantly depending on the stated purpose, making it crucial that NAMs are evaluated to ensure that they are fit for purpose for the assessment type (e.g., prioritization, classification, hazard assessment, risk assessment). Regional regulations and current acceptance of NAMs under those regulations are listed in Table 1. The OECD Guidance Document 150 provides information on standardized test guidelines for evaluating chemicals for endocrine disruption (ED) and describes what tests are available to evaluate endocrine activity and ED (OECD, 2018a). While it has been used as a basis for some regulatory guidance, it does not in itself define a testing strategy. It should be recognized that although regulatory testing requirements may vary globally, many chemicals are intended for use worldwide, and the relevance of any test across geographies (NAM or otherwise) should be a key consideration. Streamlined data packages and harmonized data templates that satisfy multiple global requirements will ensure that collected data can be broadly utilized for regulatory purposes, thereby reducing duplicative testing and resource costs, while still enabling marketing internationally (Sewell et al., 2017) and potentially reducing the need for animal testing.

## BUILDING CONFIDENCE IN NAM DEVELOPMENT, EVALUATION, AND APPLICATION

Although NAMs can be used to reduce the number of animals and resources used in toxicity testing (Parish et al., 2020), in vivo data have been typically thought to provide more realistic information on chemical hazard, especially with respect to complex biological processes and systems within the assays (like ADME). Therefore, there are still several issues to overcome before there is sufficient confidence in NAM data for these tools to be incorporated more widely in safety assessment. There is a concern for high false-negative or false-positive outcomes with NAMs, leading to a reluctance to make decisions solely based on results from these assays. However, in vivo tests may also produce false negatives because their sensitivities and specificities for detecting endocrine activity have been shown to be not as high as those of some NAMs. A separate project from this collaboration will critically review the robustness of currently available in vivo tests. Building confidence in negative outcomes from NAMs is essential to avoid unnecessary animal studies. Confidence can be increased by using multiple NAMs covering multiple pathways to ensure that there are no “gaps” in coverage. Some degree of redundancy (i.e., several NAMs covering the same molecular initiating event) may also help in consolidating a positive or negative outcome. Next, to gain regulatory acceptance, NAMs must be “validated” (appropriate definitions of validated include “the process by which the reliability and relevance of a particular approach, method, process or assessment is established for a defined purpose” [OECD, 2005]; “the process by which the reliability and relevance of a procedure are established for a specific purpose” [Balls et al., 1995]; and “the process by which the reliability and relevance of a procedure for a specific purpose are established” [National Toxicology Program Interagency Center for the Evaluation of Alternative Toxicological Methods & Interagency Coordinating Committee on the Validation of Alternative Methods, 2003]), which has its own set of challenges. For example, validation studies are usually conducted using reference test substances, which typically elicit strong negative or positive responses. However, most “real-world chemicals” tested for regulatory purposes will typically not elicit such clear effects. This has been recognized, with some OECD validation studies now including weakly acting reference chemicals to ensure that assays can cover a realistic spectrum of effect magnitudes. The USEPA is also developing and utilizing reference chemical sets with a broad spectrum of potency for validation purposes.

**FIGURE 2:**
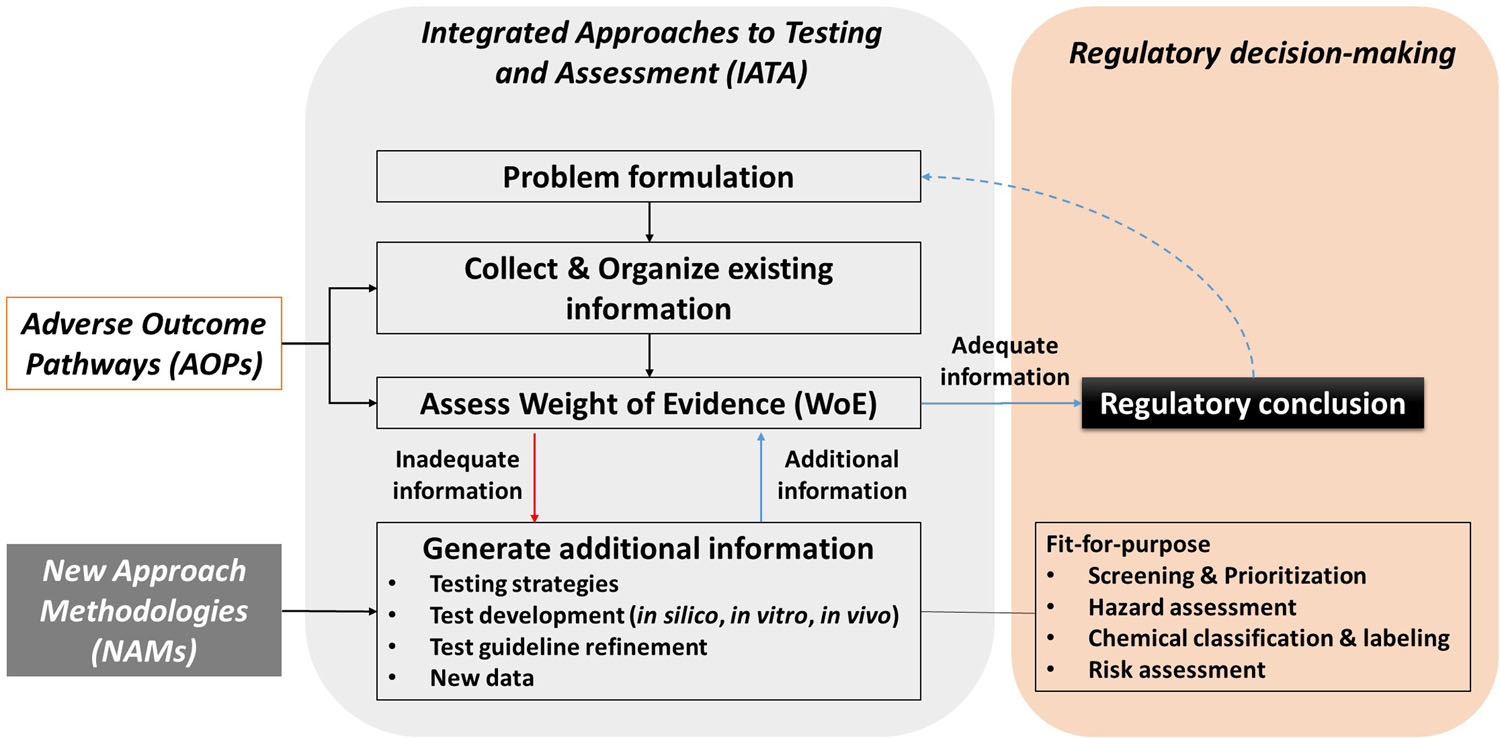
An illustration of how new approach methodologies can be integrated to support integrated approaches to testing and assessments. Modified from Organisation for Economic Co-operation and Development (2016). AOP = adverse outcome pathway; NAMs = new approach methodologies; IATAs = integrated approaches to testing and assessments; WoE = weight of evidence.

Combining data from multiple sources, such as via IATAs which use new and existing data in a flexible manner to address regulatory needs, will build confidence in NAMs. Integrated approaches to testing and assessments are widely recognized as the best regulatory practice for the application of NAMs. However, the current OECD process for IATA adoption is rigid, conservative, and lengthy (5–10 years). It typically requires that data are generated using official test guideline methods, which can take many years to obtain. Although some performance-based test guidelines are considered, there need to be faster ways to enable adoption of NAMs more broadly within regulatory agencies. Figure 2 provides a conceptual diagram regarding the integration of NAMs into IATAs.

## CURRENTLY AVAILABLE NAMs TO ELUCIDATE ED PATHWAYS AND MECHANISMS

**FIGURE 3:**
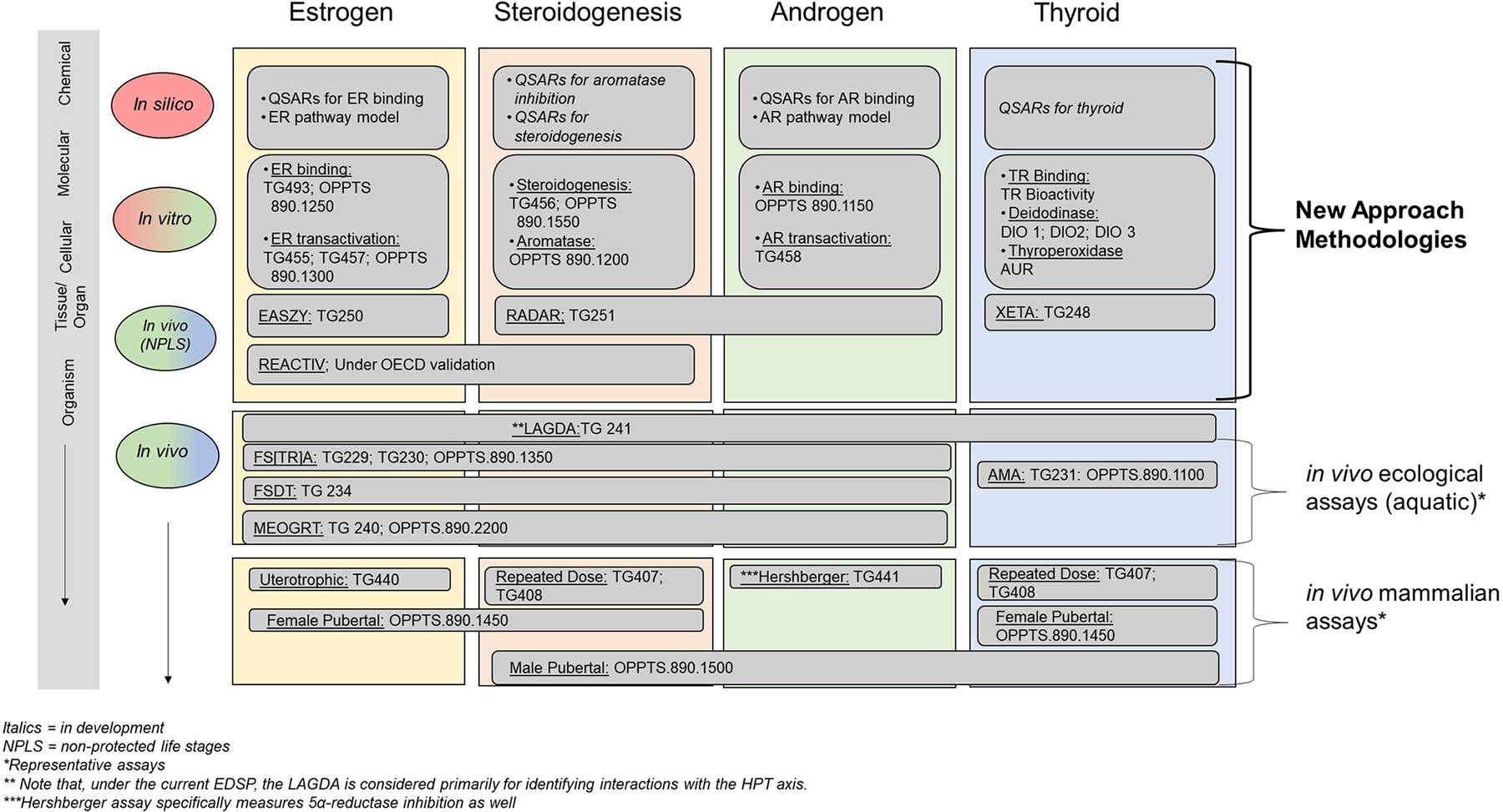
New approach methodologies and in vivo assays available to evaluate endocrine modalities. Italics indicate in development. Test guidelines: 248 (OECD, 2019), 250 (OECD, 2021b), 251 (OECD, 2022), 407 (OECD, 2008), 408 (OECD, 2018b), 440 (OECD, 2007), 441 (OECD, 2009c), 455 (OECD, 2021c), 456 (OECD, 2011a), 457 (OECD, 2012b), 458 (OECD, 2020), 407 (OECD, 2008), 493 (OECD, 2015c). *Representative assays. **Note that, under the current Endocrine Disruptor Screening Program, the LAGDA is considered primarily for identifying interactions with the hypothalamic–pituitary–thyroid axis. ***Hershberger assay specifically measures 5α-reductase inhibition as well. NPLS = nonprotected life stage; QSAR = quantitative structure–activity relationship; ER = estrogen receptor; AR = androgen receptor; TR = thyroid hormone receptor; TG = test guideline; OPPTS = Office of Prevention, Pesticides and Toxic Substances; DIO1, DIO2, and DIO3 = iodothyronine deiodinases 1, 2, and 3; AUR = Amplex UltraRed; EASZY = endocrine active substances, acting through estrogen receptors, using transgenic tg(cyp19a1b:GFP) zebrafish embryos; RADAR = rapid androgen disruption activity reporter; XETA *= Xenopus* eleutheroembryonic thyroid assay; REACTIV = rapid estrogen activity tests in vivo; OECD = Organisation for Economic Co-operation and Development; LAGDA = larval amphibian growth and development assay; FS[TR]A = fish short-term reproduction assay; FSDT = fish sexual development test; AMA = amphibian metamorphosis assay; MEOGRT = medaka extended one generation reproduction test.

This section highlights the currently available NAM tools (Figure 3): in silico approaches, in vitro assays, and eleutheroembryo-based assays. Details on each assay are available in the Supporting tables. Table 1 also demonstrates the regulatory contexts where NAMs are already being applied.

### In silico tools

In silico (i.e., computational) tools represent a growing body of NAMs with the potential to accelerate the toxicological screening process. Advantages of these methods are that they often have high speed and throughput, are low-cost, and can assess a large number of molecules with relative ease. Currently, these tools can be used as an alternative or as a supplement to in vitro and in vivo testing. Most of the available in silico models can be described as ligand-based methods or target structure-based approaches. Quantitative structure-activity relationship (QSARs) models are ligand-based methods that estimate activity based on fingerprints and molecular descriptors (Schneider et al., 2019). These tools look for structural alerts in either a particular portion of the molecule or the molecule as a whole. Examples include the OECD QSAR toolbox (qsartoolbox.org), VEGA HUB (www.vegahub.eu), or CAESAR (www.caesar-project.eu). There are QSARs for specific modalities for the endocrine system. Specifically, estrogen receptor (ER), AR, and aromatase inhibition for steroidogenesis have been well established because of interest and regulatory requirements; but models for other pathways, such as thyroid, still require significant development.

Two such QSAR models were developed by consortia of scientists from 35 international organizations led by the USEPA. The Collaborative Estrogen Receptor Activity Prediction Project evaluates chemicals for potential ER activity (Mansouri et al., 2016), and the Collaborative Modeling Project for Androgen Receptor activity (Mansouri et al., 2020) evaluates potential AR activities. Expert modelers and computational toxicology scientists used the data for approximately 1800 chemicals from the ER (Judson et al., 2015) and AR (Kleinstreuer et al., 2017) pathway models (see below, *In Vitro Tools*) to train QSAR models to predict ER and AR activity, respectively. Both of these QSARs are available through the Open Structure–activity/property Relationship App (National Toxicology Program, 2021). In addition, the USEPA’s CompTox Dashboard (Williams et al., 2017) has a “Predictions” tool (USEPA, 2022a) which allows input of a chemical structure to predict a number of toxicological endpoints including ER relative-binding affinity and ER binding.

There are also target structure-based approaches for EDCs. These investigate EDC targets using docking or more computationally intensive approaches such as molecular dynamics. Docking tools are commonly used for virtual screens for thousands of chemicals, as employed in drug discovery. There are many programs available to understand molecular docking and dynamics: A full review of these is beyond the scope of the present study (see Schneider et al., 2019, for a comprehensive summary).

While the above-described computational tools have great utility for screening and prioritization of testing efforts, most have some common limitations. Typically, the chemical space evaluated by these tools is dependent on the amount of high-quality data that are available to train the models, which is often limited. Many models are also dependent on experimental data which, in itself, can contain error and bias, in some cases leading the models to inaccurate predictions. Currently, many types of chemistries are incompatible for these models, including halogens, metals, and polymers. Chemical mixtures also prove challenging, with models overlooking potential additive or synergistic effects. From an ecotoxicological perspective, many of the existing tools are based on human data, with unknown specificity for fish or amphibians. The ADME properties are also not always incorporated into models and need to be considered (though physiologically based pharmacokinetic modeling could aid with this; Schneckener et al., 2020). Further, most computational models can only ever provide mechanistic information on the lower levels of organization in an AOP; therefore, more data will be needed to link activity detected in silico with an adverse outcome at higher levels.

The tools described above use toxicity data and/or chemical structures as their inputs. Other tools evaluate conservation where there is knowledge of targets, such as the USEPA’s Sequence Alignment to Predict Across Species Susceptibility (SeqAPASS; https://seqapass.epa.gov/seqapass/) tool. This tool provides a screening approach that allows for extrapolation of toxicity information across hundreds of species. For some model species (e.g., humans, mice, rats, and zebrafish) the USEPA has large amounts of data regarding their toxicological susceptibility to various chemicals. However, the existing toxicity data for the whole diversity of species are limited. The SeqAPASS tool extrapolates from these data-rich organisms to other unassessed species to predict their specific potential chemical susceptibility. Some chemical classes have relatively well-defined protein targets (e.g., pesticides), and those proteins are curated in the National Center for Biotechnology Information’s protein database (www.ncbi.nlm.nih.gov/protein). The SeqAPASS tool evaluates the similarities of protein structure to identify whether a protein target is present for a chemical interaction in other nontarget species. This tool has been applied to endocrine-specific targets, like the ER, the AR, and proteins in the thyroid axis and involved in steroidogenesis (Ankley et al., 2016; LaLone et al., 2018). There are advantages to tools such as SeqAPASS compared with other computational methods. For example, mixtures could be evaluated if their contents are known. Limitations relevant to SeqAPASS are related to the growing sequence information that may have poor annotation or curation and the limitations of sequence data for certain groups of species, particularly invertebrates. In addition, because the chemical’s molecular (protein) target is used to query the tool, there needs to be a better understanding (or sharing of knowledge generated in the development of chemicals) as to the chemical–protein interaction that leads to the effectiveness of the chemical.

Overall, computational methods can be used in fit-for-purpose ways as a part of a WoE assessment.

### In vitro tools

In vitro tools can provide mechanistic information and evidence on chemical perturbations via molecular initiating events and KEs in an AOP. In vitro tools can be cell-free or cell-based and, as with other NAMs, are typically used in a WoE approach.

**FIGURE 4:**
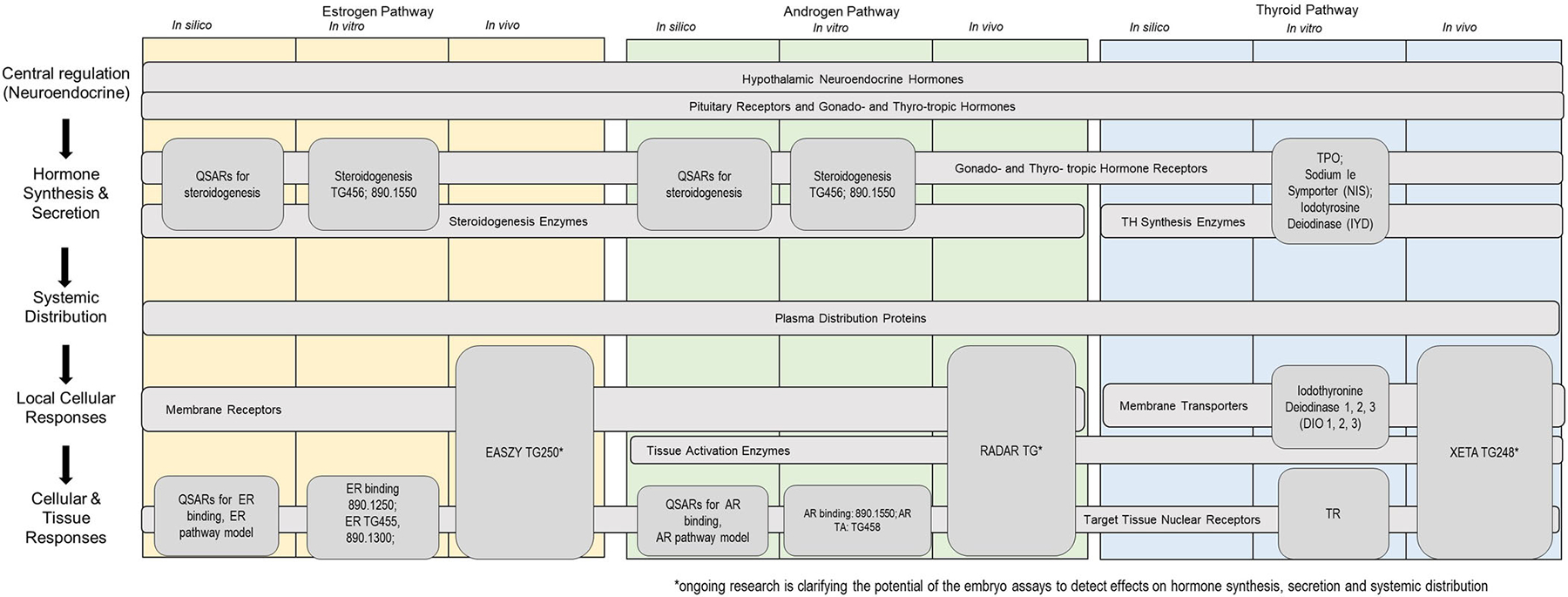
Generalized nodes with potential molecular initiating event targets across the estrogen, androgen, and thyroid pathway axes and cross-referenced with available new approach methodologies by type for measuring molecular endpoints. *Ongoing research is clarifying the potential of the embryo assays to detect effects on hormone synthesis, secretion, and systemic distribution. QSAR = quantitative structure–activity relationship; TG = test guideline; TH = thyroid hormone; TPO = thyroperoxidase; NIS = sodium iodide symporter; IYD = iodotyrosine deiodinase; ER = estrogen receptor; EASZY = endocrine active substances, acting through estrogen receptors, using transgenic tg(cyp19a1b:GFP) zebrafish embryos; AR = androgen receptor; RADAR = rapid androgen disruption activity reporter; DIO1, DIO2, and DIO3 = iodothyronine deiodinases 1, 2, and 3; TR, thyroid hormone receptor; XETA *= Xenopus* eleutheroembryonic thyroid assay.

Cell-free assays are available to measure potential endocrine interactions. While there are countless models and targets, in this review we will highlight a few assays that are currently being used or considered for screening associated with chemical safety or risk assessment (Table 1 and Figure 4). When possible, a description of the assay and the associated parameters and performance was generated and is included in the Supporting Information. There are a number of cell-free assays that have been adopted as test guidelines by either the USEPA or the OECD. The USEPA’s Endocrine Disruptor Screening Program (EDSP) Tier 1 battery of assays (USEPA, 2022a, 2022b) includes three test guidelines for cell-free assays: an AR-binding assay (rat ventral prostate cytosol, OPPTS 890.1150; USEPA, 2009b), an ER-binding assay (using rat uterine cytosol, OPPTS 890.1250; USEPA, 2009a), and an aromatase inhibition assay (human recombinant, OPPTS 1200; USEPA, 2009c).

There are also several cell-based assays adopted as test guidelines. The USEPA’s EDSP Tier 1 battery of assays includes cell-based test guidelines for ER transcriptional activation (OPPTS 890.1300; USEPA, 2009d) and steroidogenesis (OPPTS 890.1550; USEPA, 2009e). The OECD test guidelines include androgen (458, 455, 493; OECD 2020, 2021c, 2015c, respectively) and steroidogenesis (456; OECD, 2011a). There is also a yeast ER assay from the International Organization for Standardization (2018a, 2018b). The above test guidelines that are a part of the USEPA Tier 1 EDSP (see Table 1) are intended to be reviewed as a battery, which includes five in vitro tests and six in vivo tests, to determine potential estrogen, androgen, and thyroid bioactivity.

The Toxicity Forecaster (“ToxCast”) program and the Tox21 (“Toxicity Testing for the 21st Century”) federal consortium prioritize the public release of bioactivity screening data. The USEPA has developed the ToxCast program to make in vitro medium- and high-throughput screening assay data publicly available for the prioritization and hazard characterization of thousands of chemicals. ToxCast contains a variety of assay technologies to evaluate the effects of chemical exposure on diverse biological targets from distinct proteins to more complex cellular processes, including all Tox21 assays. The USEPA’s CompTox Chemicals Dashboard (https://comptox.epa.gov/dashboard) integrates physicochemical, environmental fate and transport, exposure, usage, in vivo toxicity, and in vitro bioassay data (i.e., ToxCast) for over 870 000 chemicals (Williams et al., 2017). A subset of ToxCast methods and data from this effort is directly relevant to screening for endocrine activity. For example, ToxCast has 18 high-throughput assays based on the molecular events associated with ER activation (e.g., binding, dimerization, transcription, and translation), and the data from these assays were assessed for 1800 chemicals to build a statistical model called the *ER pathway model* (Judson et al., 2015). Similarly, ToxCast has 14 high-throughput assays based on the molecular events associated with AR activation, and the data from 11 assays were assessed for the same set of 1800 chemicals to build the *AR pathway model* (Judson et al., 2020; Kleinstreuer et al., 2017). These models can also provide a measure of potency based on consensus models, where relative potency is scored against the bioactivity of known potent substances (e.g., 17β-estradiol). The data for each of the ER and AR high-throughput assays and scores for both the ER and AR pathway models are available on the Chemicals Dashboard. In 2015, the USEPA published a *Federal Register* notice seeking comment on using the ER pathway model as a validated alternative for multiple Tier 1 assays (USEPA, 2015a). Very recently, the USEPA published a white paper supporting the validation of these models as alternatives for EDSP Tier 1 screening (USEPA, 2023). The ToxCast ER and AR high-throughput assay data were reassessed to demonstrate that subsets of assays can provide close to the same level of predictivity as the full assay sets listed above for ER agonism (Judson et al., 2017), AR agonism, and AR antagonism (Judson et al., 2020).

The Chemicals Dashboard also contains ToxCast data from assays targeting multiple thyroid hormone receptors (TRs; Paul-Friedman et al., 2019), thyroperoxidase activity (Paul Friedman et al., 2016), and steroidogenesis (HT-H295R; Haggard et al., 2017). New ToxCast assays for a variety of thyroid pathways are in development. These include the iodothyronine deiodinase 1 (DIO1) inhibition assay (Hornung et al., 2017), the DIO2 inhibition assay (Olker et al., 2018), the DIO3 inhibition assay (Olker et al., 2018), the iodotyrosine deiodinase inhibition assay (Olker et al., 2021), and the sodium iodide symporter inhibition assay (J. Wang et al., 2019).

The currently available in vitro tools have common strengths in that they are quick to perform, many have been adapted to be high-throughput, and they provide specific mechanistic information on potential endocrine activity. The cell-based in vitro assays also share some common strengths, utilizing immortalized and commercially available cell lines, which are easier to acquire and handle than primary cells and do not need to be sourced from animals. They also can give some idea of potency based on response or binding (Asnake et al., 2019). However, the immortalized cell lines used in the above assays are almost exclusively derived from tumors and are prone to the *Warburg effect* (increase in the rate of glucose uptake and preferential production of lactate, even in the presence of oxygen; Oliveira et al., 2015), which could be a complication for predicting responses to endogenous cells, receptors, and enzymes. Not all modalities and mechanisms are fully covered by existing assays, and there are few that are specific for fish or amphibians. Some cell-free assays require isolation of biological material from animal organs (e.g., the AR-binding assay uses rat ventral prostate cytosol). Using biological material derived from animals increases cost and effort; however, those organs have endogenous expression of a mix of receptors (e.g., uterus expresses ERα and ERβ) that may not be found in immortalized cell lines. In addition, receptor-binding assays cannot distinguish if a chemical is an agonist or antagonist, and most in vitro assays would likely not be able to capture responses from life stages when an organism would be more susceptible to the effects of an EDC.

### Eleutheroembryo assays

Eleutheroembryo assays have been developed in amphibians and fish with the specific purpose of detecting endocrine activity. The amphibian *Xenopus laevis* is used in the *Xenopus* eleutheroembryonic thyroid assay (XETA; OECD test guideline 248; OECD, 2019). Medaka (*Oryzias latipes*) and zebrafish (*Danio rerio*) are fish species for which eleutheroembryo assays have been developed to investigate estrogenic (endocrine active substances, acting through estrogen receptors, using transgenic tg[cyp19a1b:GFP] zebrafish embryos; OECD test guideline 250 zebrafish eleutheroembryos; OECD, 2021b; rapid estrogen activity tests in vivo [REACTIV] assay, medaka eleutheroembryos) and androgenic (rapid androgen disruption activity reporter [RADAR] assay; OECD test guideline 251 medaka eleutheroembryos; OECD, 2022) activities. Both the REACTIV and RADAR assays also cover downstream steps (5α-reductase and aromatase) of steroidogenesis. These assays use organisms in the eleutheroembryonic stage that are not capable of independent feeding. At this stage the eleutheroembryos are not considered capable of experiencing pain, distress, suffering, or lasting harm (European Food Safety Authority [EFSA], 2005). However, it is important to note that these eleutheroembryo assays do not necessarily use fewer individuals compared with other OECD Level 3 mechanistic assays (e.g., test guidelines 231, 229; OECD, 2009b, 2012a, respectively).

Currently, the available eleutheroembryo assays do not allow for the identification of specific endocrine targets because they are whole organisms which may include multiple mechanisms by which an endocrine active substance can act. By design, the assays are based on the use of genetic constructs that combine the promoter of an endocrine receptor and the reporter gene of a green fluorescent protein (GFP). The assays measure the capacity of a substance to activate or inhibit the transcription of the genetic construct, which can happen via different mechanisms. For example, the XETA uses the promoter of the thyroid hormone/basic leucine zipper (TH/bZIP) gene, the expression of which is regulated directly at the onset of metamorphosis by TH (Furlow & Brown, 1999). The transcription of TH/bZIP-GFP is regulated either directly through binding to the TR or modifying the binding of thyroid hormones or indirectly by modifying the amount of hormone available to activate the receptor. Similarly, the REACTIV and RADAR assays can detect substances that directly bind the ER or AR (i.e., ER and AR agonists), respectively, and substances that affect the enzymes of the downstream steps of steroidogenesis (i.e., 5α-reductase and aromatase). It is acknowledged that organisms in the eleutheroembryonic stage do not produce (sufficient) endogenous hormones like thyroid hormones or steroids. However, recent studies with fish embryos to assess the effects of the potent estrogen 17α-ethynylestradiol have shown that nonregulated early life stages respond in a manner and with a sensitivity that are similar to sexually mature fish (i.e., production of vitellogenin; Alcaraz et al., 2021). The detection of antagonistic effects, therefore, requires additional treatments where an exogenous hormone is coexposed in the test design.

In the absence of in vitro data, the eleutheroembryo assays can only inform on the potential for a test substance to interact directly or indirectly with an endocrine axis via various mechanisms. The level of specificity toward the specific endocrine mechanisms is, however, greater for the eleutheroembryo assays than for their in vivo screens (amphibian metamorphosis assay [AMA]; OECD test guideline 231; OECD, 2009b) or the fish short-term reproduction assay (FSTRA; OECD test guideline 229; OECD, 2012a), where nonendocrine mechanisms can also induce the response of most (if not all) measured apical endpoints (i.e., development or reproduction).

There are still uncertainties concerning the metabolic capabilities of eleutheroembryos. The early developmental stages covered have metabolic enzyme expression that is different from that of more developed and adult individuals. Current limitations to the use of eleutheroembryonic assays in regulatory endocrine assessment are mainly related to establishing applicability domains for the mechanisms covered and capacity and experience in laboratories, combined with limited regulatory acceptance. European regulatory authorities are becoming more familiar with the use of the XETA in EDC assessments. The European Chemicals Agency (ECHA) and EFSA recently amended the guidance for the identification of endocrine disruptors in the context of Regulations (EU) No. 528/2012 and (EC) No. 1107/2009, with an annex describing the conditions of use of the XETA as a mechanistic assay to detect thyroid active substances (Arena & van der Linden, 2021). In this document, the XETA is recommended as an alternative to the AMA under certain conditions. The potential of the XETA to predict the outcome of the AMA has been recently investigated using 22 chemicals that have been tested in both assays, showing a high concordance between the outcomes of the two assays for thyroid active and inactive chemicals (D. Du Pasquier, Laboratoire Watchfrog, F-91000 Evry, France, unpublished data). The forthcoming publication of fish eleutheroembryo assays as OECD test guidelines will further contribute to promoting the regulatory use of this type of assay as NAMs for assessment of endocrine activity.

## INTEGRATION AND APPLICATION OF NAMs

### NAMs and AOPs

Despite some of the limitations related to NAMs described above, these tools have great promise for integration into the AOP framework. Within the AOP framework, NAMs primarily fit to the “left side” (Figure 1), providing endocrine activity information at the molecular, cellular, and tissue levels. Eleutheroembryo assays can provide information on the whole organism level, although they may be limited by developmental stage and metabolic competence. Figure 4 shows the generalized nodes, neuroendocrine signaling, hormone synthesis, systemic distribution, and cellular and tissue responses, of the endocrine pathways for the estrogen, androgen, and thyroid hormone axes. Within each of these nodes, there are different mechanisms which could be targets for chemical perturbation. The figure shows the NAMs currently available or in development at each of the pathway nodes and can be used to help identify gaps in the biological coverage and potentially inform future NAM development, especially for the assessment of thyroid activity.

There are currently 20 endocrine AOPs registered at the OECD, of which three are endorsed, seven are under review, and 10 are under active development (Table 2). These AOPs cover the estrogen, androgen, thyroid, and steroidogenesis modalities in mammals and fish. Various NAMs are deployed as the detection/quantification methods for measuring the molecular initiating events or early KEs in these AOPs (Table 2). Many NAMs can generate temporal and dose–response data (e.g., the US Tox21 quantitative high-throughput screening program), not only providing empirical support for WoE assessment of the AOPs but also forming the basis for the construction of quantitative prediction models (i.e., quantitative AOPs) in the future. Conversely, connecting to AOPs may facilitate the development of NAMs toward regulatory acceptance because AOPs serve as bridges to link the molecular/cellular effects measured by NAMs to adverse effects of regulatory concern. However, a current weakness is the difficulty in connecting these molecular initiating events and KEs to adverse effects at the organism and population levels aligned to the protection goal of most environmental assessments (Lagadic et al., 2020).

To address this challenge, putative AOPs may be developed where molecular/cellular responses to a chemical exposure are experimentally determined using in silico and in vitro assays, which are then linked to KEs leading to an adverse outcome. For the environmental endocrine assessment, these adverse outcomes will need to be empirically demonstrated as directly relevant to the population, or the AOP will need to incorporate an additional modeling step to estimate if the individual-level effects translate to the apical assessment endpoints at the population level. Once constructed, these AOPs may be used qualitatively to evaluate chemicals for potential toxicity and, in some cases, quantitatively to build mechanistic models for predicting dose–response relationships and risk estimates (El-Masri et al., 2016). However, the application of AOPs necessitates the extrapolation of in vitro data to in vivo doses, which are converted to external exposure levels where regulatory limits are set. Such in vitro to in vivo extrapolations (IVIVE) help interpret the biological plausibility of in vitro concentrations that induce effects by placing them into the context of tissue concentrations. However, linking tissue concentrations to external doses requires an understanding and characterization of ADME for each chemical (Cohen Hubal et al., 2019; Strikwold et al., 2013). Species differences in ADME also need to be considered (e.g., ADME in humans is not the same as ADME in fish and/or amphibians).

### NAMs and IATAs

One barrier to the adoption of some NAMs in certain regulatory contexts is the lack of harmonized, internationally recognized guiding principles on how and when to apply and interpret these new approaches and data for decision-making (Webster et al., 2019). Integrated approaches to testing and assessments are strategies that use new and existing data in a flexible manner to address regulatory needs (Figure 2). To gain acceptance of NAMs, multiple programs, including Accelerating the Pace of Chemical Risk Assessment (Kavlock et al., 2018), EU ToxRisk (Moné et al., 2020), and the OECD IATA Programme, are using case studies to explore how to apply and interpret NAMs in a decision-making context. This should lead to more widespread acceptance of these tools.

New approach methodologies integrated into AOPs, IATAs, and other WoE paradigms hold promise for more efficient and timely chemical assessments based on sound. However, they are not one-to-one replacements for in vivo tests and should be used in a battery and applied in a WoE approach. If incorporated in this way, NAMs could be used to simultaneously improve the science of endocrine activity and disruption assessment by providing a better mechanistic understanding compared with animal data alone.

## INCREASING REGULATORY ACCEPTANCE OF NAMs

There has been a consistent stream of NAM development within the fields of both human health and ecological safety assessment over the past decade, sparked by efforts to reduce the need for more resource-intensive animal testing. In turn, the shift toward a more mechanistic focus and the increase in the use and application of AOPs have provided the platform and framework to support the further development, interpretation, and use of NAMs. Particularly in the endocrine space, robust knowledge of the estrogen, androgen, thyroid, and steroidogenesis pathways and a high degree of conservation across species afford a unique opportunity to utilize and apply NAMs within regulatory contexts but also in ecological risk assessments more broadly. While not exhaustive, several short- and longer-term priorities to help facilitate the regulatory use of NAMs are discussed below.

### Short-term

#### Define and increase the applicability domains of available NAMs:

For assays already well developed and in use, approaches to expand their use and establish agreed chemical and taxonomic applicability domains should be investigated so that information can be incorporated into regulatory guidance. For example, greater application of NAMs could be encouraged by use of performance-based testing guidelines. Greater latitude in the experimental design is allowed if certain “performance” criteria are still met. This means that laboratories with analogous methods can still apply their models within a test guideline framework that in turn should be more broadly acceptable to regulatory authorities and study sponsors. Recent experience has also demonstrated the need for greater clarity around applicability domains under which the data can be considered reliable for regulatory decision-making. One recent example is the appendix (Arena & van der Linden, 2021) to the ECHA/EFSA guidance document for the application of the XETA test guideline for plant protection products in the European Union. This appendix has increased regulatory clarity for registrants to decide when the XETA assay is appropriate over the performance of the in vivo AMA. This highlights the importance of the international validation program to ensure that there is clarity on the applicability domains reliably covered (or not) as demonstrated in the validation exercise. These activities all work to increase confidence within regulatory authorities, which in turn provides reassurance to registrants that NAM approaches are likely to be accepted in place of in vivo approaches or at least reliably inform when in vivo studies are truly necessary.

#### Further develop NAMs for the thyroid pathway:

The thyroid modality remains an area where, despite the development of numerous NAM assays for molecular initiating events, there are no validated in vitro test guidelines (Noyes et al., 2019) or OECD conceptual framework Level 2 assays. Development of additional NAMs to evaluate thyroid effects is needed. At present, the use of in vitro data beyond screening and prioritization for thyroid modality is challenged by the complexity of potential thyroid-related adverse outcomes and the limited knowledge of mechanistic processes controlling such responses. There are emerging tools like those described above, as well as the Danish QSAR Database which includes thyroid disruption models (Rosenberg et al., 2017). Additional research into the role of the thyroid in fish and amphibians may be needed before NAM tools can be developed (Knapen et al., 2020).

#### Increase understanding of ADME processes related to NAMs:

Incorporating ADME data with NAMs will increase scientific confidence. Examples of recent work to do this include the USEPA’s development of the Alginate Immobilization of Metabolic Enzymes platform to include metabolic competence in an ER transactivation assay (Deisenroth et al., 2020) and additional work that introduces metabolic capacity into the cells by performing transfections with modified mRNAs (DeGroot et al., 2018). Quantitative IVIVE (QIVIVE), which uses concentration responses from in vitro toxicodynamic or toxicity assays along with in vitro toxicokinetic measures (e.g., plasma binding) as inputs to a physiologically based pharmacokinetic model to derive circulating plasma concentrations (Bell et al., 2018; Wetmore, 2015), is being used to predict in vivo toxicity. Quantitative in vitro to in vivo extrapolation is increasingly being used to predict toxicity with human (Beames et al., 2020; Clewell et al., 2019; Paul Friedman et al., 2020; Shi et al., 2020) and rat (Honda et al., 2019; Scholze et al., 2020) models; however, the process has not yet been applied to fish or amphibian endocrine test endpoints. Considerations for implementation of QIVIVE to a fish model include appropriate in vitro toxicity assays for fish, in vitro toxicokinetic assays, and physiologically based pharmacokinetic models capable of calculating external aquatic exposure concentrations from predicted plasma concentrations. Assays for plasma binding (Henneberger et al., 2020) and intrinsic metabolic clearance (Black et al., 2021; Nichols et al., 2018) have been developed for representative fish species, as have detailed models (Brinkmann et al., 2014; Grech et al., 2019; Nichols et al., 2004, 2018; Stadnicka et al., 2012). Many components necessary for the implementation of QIVIVE for fish models are available; however, more toxicokinetic data are needed to develop the requisite data sets for comparison with fish endpoints relevant for screening or testing for ED.

#### Curate high-quality in vivo data in standardized formats:

For the foreseeable future, the performance of NAMs will be benchmarked against the results and decisions that have been made using in vivo study data. Therefore, it is imperative to make high-quality in vivo data accessible for the relevant comparisons to be made in assessing NAM performance. Despite the wealth of in vivo toxicity data that exist, finding, collecting, and assessing these data can be challenging. Many publicly available regulatory submissions would require manual, time-consuming document extraction; and reports may not be made available immediately or may be redacted because of the inclusion of proprietary information. To facilitate the incorporation of NAMs into the EDC regulatory space, effort needs to be made to map what data exist and ensure that the data are available in a useable format. Advances in data curation science and machine learning data tools hold great promise in addressing this challenge. Another difficulty lies in the lack of data standardization that exists across sectors, regions, and individual studies. Looking forward, facilitating data interoperability and usage will require harmonized language and data templates, preferably located within a centralized international database. A parallel project mentioned previously assessing the robustness of the current in vivo assays has established databases of regulatory data for the AMA, medaka extended one generation reproduction test (OECD test guideline 240; OECD, 2015a) and FSTRA (OECD test guideline 229; OECD, 2012a). These databases could be leveraged to enable the relevant comparisons.

#### Continue to “map” NAMs using the AOP framework:

Identifying where various NAMs fit along an AOP facilitates clear and transparent communication across the scientific and regulatory communities about their use and potential application. In addition, it allows anchoring of various NAMs to adverse outcomes and clearly identifies where there are gaps in method development and/or quantitative understandings across KEs. The continued mapping and usage of the AOP framework should be encouraged.

#### Develop case studies:

Where available, case studies for chemicals and chemical classes with existing NAM and in vivo data should be analyzed to compare the differences in conclusions from assessments. This could support NAMs being used for risk or hazard assessment instead of just prioritization or screening. These case studies could incorporate paradigms like IATAs or AOPs to use multiple evidence streams in a WoE analysis for evaluation. This type of work is being initiated within this collaboration.

#### Consistent dialogue across the scientific and regulatory communities:

Engaging regulatory authorities early on in defining data needs is critical and allows greater provision of opportunities where data generated using NAMs can be submitted and assessed in parallel with traditional in vivo data to build confidence. Such an approach has been encouraged in data requests on substances undergoing evaluation or reevaluation at the Joint FAO/WHO Meeting on Pesticide Residues, with the output offering to “evaluate without prejudice, in parallel, any data generated using emerging methods that in the view of sponsors could substitute for information obtained using conventional testing methods” (Food and Agriculture Organization & WHO, 2021). With time and experience, it could be assessed whether both in vitro and in vivo approaches are always needed to make appropriate decisions. The normalization of including NAM data within submissions would also gain acceptance among regulatory scientists who interpret these different data sources.

### Longer-term

#### Continued funding and allocation of resources to support NAMs:

Consistent and adequate allocation of resources is needed to validate and establish which NAMs (or battery of NAMs) are best able to reduce or replace and improve on in vivo testing approaches. Validation work tends to lack specific funding mechanisms and is not viewed as being as “exciting” as basic research, but it is the most critical step toward regulatory use and application. Validation will encourage the use and uptake of these methods as well as opportunities to incorporate additional endpoints into these assay batteries, which could reduce overall time and costs.

#### Development of quantitative linkages across KEs: Generally, AOPs are described in qualitative terms:

They usually do not provide an understanding of quantitative relationships between the molecular initiating events, subsequent KEs, and the ultimate adverse outcome (Sewell et al., 2018). The important, and not yet met, requirement is that AOPs reflect the thresholds required for progression of a defined biological perturbation to a specific toxicity. In all areas, there may be some data regarding the thresholds characterizing KE relationships that already exist and may include information on dose–response relationships and temporal concordance between KEs. However, often these data are not appropriately captured or made available in a readily accessible form. If supporting evidence for KEs and KE relationships was suitably annotated with dose and time information, then it would be possible for temporal and dose concordance between KEs to be inferred computationally based on Bradford-Hill considerations (Becker et al., 2015). There has been some notable movement in this area to date (Conolly et al., 2017; Villeneuve et al., 2021), but more needs to be done.

#### Further development of new technologies and approaches:

Continued support of methods that are under development and have potential to support quantitative AOP development and application in the ecological space (for ED and otherwise) is needed. A brief description of several technologies that are in development and could be used for endocrine modalities is provided below. This is not meant to be exhaustive.

##### High-content imaging-based high-throughput phenotypic profiling:

Also known as *cell painting*, high-content imaging-based high-throughput phenotypic profiling is a NAM that has mostly been piloted in human cells (Ljosa et al., 2013). As opposed to targeted in vitro assays, profiling assays collect multiplexed measurements of different features and evaluate the global response of intact cells to chemicals. For toxicology, it is currently being used at the USEPA to compare the results of in vitro studies to values from existing mammalian toxicity studies (Nyffeler et al., 2020). More work needs to be done to gain confidence in this method for human cells before applying to ecotoxicology, specifically for endocrine assessment.

##### Transcriptomics experiments:

Transcriptomics experiments are routinely run for many chemicals, but there remains difficulty interpreting pathway and/or adverse effects associated with differential gene expression (Pain et al., 2020). Greater investment in sequencing tools and genome annotation could aid the reduction of resource-intensive in vivo studies by in vitro replacements or refinement of experiments, resulting in more mechanistic information. This would also support advances in species extrapolation and better understanding of species differences. Transcriptomic assays provide mechanistic information on the specific KEs triggered by a compound but also reflect a whole-organism response that could ultimately replace currently required confirmatory higher-tier in vivo testing. While transcriptomics shows great promise and is increasingly included as supplementary information in support of chemical hazard assessment, more work is needed to support the adoption of these tools, in particular for EDC-specific outcomes. Below are some examples:

- In addition to pathway analyses, there is interest in developing transcriptomic-based points of departures. The HESI’s Emerging Systems Toxicology for Assessment of Risk Committee is currently exploring the use of transcriptomic points of departures for mammalian studies for chemicals (Johnson et al., 2022). Regarding ecotoxicity assessments, recent research has shown that transcriptomic points of departure from short-term exposure studies are protective of chronic effects for diverse fish species exposed to estrogenic chemicals (Alcaraz et al., 2021; Pagé-Larivière et al., 2019). New, user-friendly, and intuitive tools such as FastBMD (https://www.fastbmd.ca) now facilitate the derivation of transcriptomics benchmarks.
- Building on the potential for transcriptomics and other approaches to support cross-species extrapolation, a multisector consortium aims to improve the International Consortium to Advance Cross-Species Extrapolation in Regulation (LaLone et al., 2021).
- There have also been efforts to develop and standardize reduced transcriptome panels that are representative of diverse biological pathways of interest from a toxicological perspective (Soufan et al., 2019; P. Wang et al., 2018), One such example is EcoToxChips, which uses early, nonprotected vertebrate embryonic life stages (birds, amphibians, and fish) in combination with an intuitive and standardized bioinformatics platform (EcoToxXplorer.ca) for chemical prioritization and possible hazard assessment (Basu et al., 2019; Soufan et al., 2019).

##### Organ on a chip:

These assays utilize three-dimensional microfluidic cell culture to simulate an entire organ (Low et al., 2021). These models have been growing in popularity and have been used in the drug discovery process and for toxicology research. For ecotoxicology, there are various models including Fish-on-a-Chip, which uses microfluids to imitate zebrafish-based research (Yang et al., 2016), and Fish-Gut-on-Chip, which reconstructs the fish intestinal barrier (Drieschner et al., 2019). Work needs to be done to apply these tools to endocrine modalities. While these technologies could reduce the need for in vivo testing, limitations include the inability to represent the whole organism organ level because these are only organ-specific functions and do not capture organ-to-organ communication via endocrine pathways. The metabolic ability of these systems varies, as does their ability to capture organ-to-organ communication via endocrine pathways. Multiorgan systems are undergoing evaluation using combinations of up to three organ systems in other research areas however, and this type of approach may support use of organ-on-a-chip for endocrine assessments.

#### Exploration of other pathways and applications:

Endocrine pathways other than estrogen, androgen, thyroid, and steroidogenesis often lack NAMs (e.g., aryl hydrocarbon receptor, peroxisome proliferator–activated receptors, and glucocorticoid signaling; Martyniuk et al., 2021). In general, many of the concepts in the present study could apply to other modalities and other mechanisms of toxicity (e.g., neurotoxicity, genotoxicity) that are being explored via AOPs. Targeted assays could be designed for the modalities and/or combinations with nontargeted techniques could aid in the replacement and reduction of resource-intensive in vivo tests for these pathways. However, it is important to keep in mind the environmental protection goals (e.g., a refined assay for the ER modality that would capture the most important molecular initiating events of the ER axis applicable to population-relevant adverse outcomes). As with all toxicity testing, mixtures are difficult to assess. To deal with mixtures, tools need to be improved or developed to be more representative of actual organisms and environmental conditions, and cumulative exposures need to be considered for endocrine assessment.

## CONCLUSIONS

New approach methodologies can be utilized to both reduce the number of animals needed to assess a chemical’s endocrine potential and simultaneously improve the science of endocrine activity and disruption assessment. While NAMs are currently used in some regulatory assessments and when data are available, advances need to be made to be able to move away from in vivo testing to a fit-for-purpose test battery of NAMs. New approach methodologies should be integrated as part of a WoE approach for hazard or risk assessment using the AOP framework. The application and use of NAMs within a WoE approach may satisfy outstanding data needs, testing requirements, or protection goals. Immediate next steps to advance adaptation of NAMs in regulatory contexts include performing case studies on compounds that have been evaluated with NAMs and in vivo methods in a regulatory context and comparing the outcomes.

## Supplementary Material

Supplement1

## Figures and Tables

**TABLE 1:** Current regulatory requirements and opportunities for new approach methodologies related to endocrine‐disrupting chemical

Region	Substance type	Legal basis	Requirements for endocrine testing using fish or amphibian models	Opportunities for NAMs

Europe	Plant protection products— active substances	Commission regulation (EU) 2018/605 amending Annex II to Regulation (EC) No. 1107/2009 by setting out scientific criteria for the determination of ED properties	OECD Conceptual Framework (OECD, 2018a) toolbox adopted. According to the Commission Delegated Regulation (EU) No. 2017/21003 and Commission Regulation (EU) No. 2018/605, the conclusions as to whether the ED criteria are met need to be drawn separately with respect to humans and nontarget organisms. The assessment strategy aims at making the most efficient use of the available data set to reach a conclusion. It is recommended to strive for a conclusion on the ED properties regarding humans and, in parallel, using the same database, to strive for a conclusion on mammals as nontarget organisms. Only where, based on this assessment, the criteria are not met for mammals as nontarget organisms would the assessment need to proceed by considering fish and amphibians because these are the taxa where standardized test methods and knowledge on how to interpret the results are available. Information on other taxa (e.g., birds and reptiles) should be considered if available (ECHA & EFSA, 2018).	Assessment of the mammalian data set includes the following in vitro assays: estrogen: OECD TG 455 (OECD, 2021c), OPPTS 890.1300 (USEPA, 2009d), and ToxCast ER Bioactivity Model Data; androgen: OECD TG 458 (OECD, 2020); steroidogenesis: OECD TG 456 (OECD, 2011a), OPPTS 890.1550 (USEPA, 2009e); EU B.57, OPPTS 890.1200 (USEPA, 2009c). In ECHA & EFSA (2018), eleutheroembryo assays (XETA, EASZY, RADAR assay) mentioned as Level-3 assays relevant for the evaluation of endocrine activity.
	Biocidal products—active substances	Commission Delegated Regulation (EU) 2017/2100 setting out scientific criteria for the determination of ED properties pursuant to Regulation (EU) No. 528/2012	Specific studies in fish may include a MEOGRT (OECD, 2015a) or a fish life-cycle study covering all the “estrogen, androgen or steroidogenesis-mediated” parameters foreseen to be measured in OECD TG 240. Specific studies in amphibians may include a LAGDA (OECD, 2015b). These studies do not need to be performed if endocrine activity is sufficiently investigated (i.e., a test according to OECD TG 229 [OECD, 2012a], 230 [OECD, 2009a], and 231 [OECD, 2009b] is available) and there is no indication that the substance has endocrine activity or effects potentially related to endocrine activity.	
	Industrial chemicals	Regulation (EC) No. 1907/2006 concerning REACH	EDs identified as substances of very high concern or equivalent level of concern are included in the candidate list and are subject to authorization/restriction processes. Currently (2022), proposals for standard information requirements are being discussed.	
	Human pharmaceuticals	Regulation (EC) No. 726/2004	Currently (2022), the European Medicines Agency's Committee for Medicinal Products for Human Use guideline (EMEA/CHMP, 2006) specifies that environmental risk of certain compounds, including endocrine-active ones, needs to be addressed irrespective of exposure. Some outlined guidance on testing approaches is also available in the accompanying Q&A document. A draft revision of the guidance released in 2018 retains the requirements for tailored risk assessment for endocrine-active substances and makes more specific recommendations for mechanism of action-specific testing, including OECD TG 229 (OECD, 2012a), 230 (OECD, 2009a), 234 (OECD, 2011b), 240 (OECD, 2015a), and 241 (OECD, 2015b).	
	Veterinary pharmaceuticals	Regulation (EC) No. 2001/82/EC	Required studies do not currently include studies specifically designed to identify endocrine-mediated effects.	Information from mechanism of action, mammalian toxicology, or open literature studies can inform requests for additional studies.
United States	Pesticides, pesticidal formulation inert chemicals, and environmental contaminants found in drinking water to which a substantial population is exposed	Food Quality Protection Act 1996. EDSP 1998 *Federal Register Notices* • August 11, 1998: EDSP • December 28, 1998: EDSP Statement of Policy	EDSP uses a two-tiered approach: Tier I • OPPTS 890.1100: Amphibian metamorphosis assay (USEPA, 2009f) • OPPTS 890.1350—Fish short-term reproduction assay (USEPA, 2009g) Tier II • OPPTS 890.2200: MEOGRT (USEPA, 2015b) • OPPTS 890.2300: LAGDA (USEPA, 2015c) The 52 pesticides on List 1 have undergone Tier I screening, and no test orders for Tier II testing have yet been issued. List 1 includes chemicals that the USEPA selected based on exposure potential.	The USEPA is currently focusing on the pivot strategy of developing and implementing NAMs as alternatives. The goal is to develop a set of “nonanimal” high-throughput assays and computational bioactivity models as alternatives for all the assays in the current Tier 1 screening battery. In vitro consensus approaches have been proposed by the USEPA for ER (USEPA, 2015a) and AR activity (USEPA, 2017; “ER/AR pathway models”).
	Human pharmaceuticals	US: National Environmental Policy Act of 1969 Code of Federal Regulations Part 25—Environmental Impact Considerations	The US FDA has issued both a general guidance for environmental risk assessment (FDA, 1998) and a Q&A for endocrine-active substances (FDA, 2016). Neither of these request specific studies, but applicants are encouraged to request to consult the agency prior to submission.	
Japan	Chemical substances	Japan Chemical Substance Control Law (2011) Current strategic program EXTEND 2017	The Japanese strategy employs a two-tiered approach*: Tier I • Medaka ERα reporter gene assay • Medaka ARβ reporter gene assay • OECD TG 231 (OECD, 2009b) • Fish screen (i.e., OECD TG 229 [OECD, 2012a] or 230 [OECD, 2009a]) • Juvenile medaka anti-androgen screening assay (under development) Tier II • OECD TG 241 (OECD, 2015b) • OECD TG 240 (OECD, 2015a)	• Medaka ER a and *b* reporter gene assays • *Xenopus tropicalis* thyroid receptor *b* reporter gene assay
Canada	Pesticides		The Pest Management Regulatory Agency considers toxicological studies that investigate the ED potential of pesticides as part of pesticide evaluations. For chemicals that exhibit ED properties or that induce adverse biochemical and/or cellular changes, the agency considers effect(s) resulting in a measurable holistic endpoint (e.g., mortality, reproduction, growth development, behavioral) to assess risk to nontarget organisms at environmentally relevant concentrations. It does not currently require studies evaluating specific ED effects for the premarket registration of new chemicals under the Pest Control Products Act, but available endocrine data are considered in risk assessments.	The Pest Management Regulatory Agency is open to considering any applicant rationale incorporating NAM results as a hazard flag for pesticides. It does not routinely use or receive NAM data for regulatory purposes. If NAM results were to flag an ED concern and exposure is expected, the substance could be considered as a candidate for additional data gathering. New approach methodology studies can impart valuable insight into the mechanistic toxicity of a chemical at the molecular and cellular levels; however, these effects may not necessarily translate into observable adverse effects at the organism level. Toxicity results of a mechanistic nature, therefore, are typically used qualitatively in the agency’s risk assessments.
	Chemical substances		Endocrine tests are not presently a regulatory requirement under the Canadian Environmental Protection Act, 1999; but available endocrine data are considered and can be a driving factor in risk assessments.	Modes and mechanisms of action considerations, including endocrine-active substances, have been applied to ecological assessments (Okonski et ah, 2021). The specific role for a given
	Human pharmaceuticals		Safety, quality, and efficacy of pharmaceuticals are evaluated under the Food and Drugs Act.	NAM will dictate both the types of data and the level of confidence needed to support its integration. For example, as replacements for apical, in vivo endpoints, NAMs would need to generate reliable, quantitative values to allow the estimation of predicted-no-effect concentrations, while NAMs intended to flag specific modes of action can be integrated through the application of additional assessment factors during predicted-no-effect concentration derivation. Canada’s Ecological Risk Classification of organic substances approach under the Chemicals Management Plan utilizes WoE approaches which incorporate in silico flags.

NAM = new approach methodology; EC = European Commission; ED = endocrine disruption/disruptor; OECD = Organisation for Economic Co-operation and Development; ECHA= European Chemicals Agency; EFSA = European Food Safety Authority; XETA = Xenopus eleutheroembryonic thyroid assay; EASZY = endocrine active substances, acting through estrogen receptors, using transgenic tg(cyp19a 1 b:GFP) zebrafish embryos; RADAR = rapid androgen disruption adverse-outcome reporter; MEOGRT = medaka extended one-generation reproduction test; TG =test guideline; LAGDA = larval amphibian growth and development assay; REACH = Registration, Evaluation, Authorisation and Restriction of Chemicals; Q&A = question and answer; EDSP= Endocrine Disruptor Screening Program; OPPTS = Office of Prevention, Pesticides and Toxic Substances; USEPA = US Environmental Protection Agency; ER = estrogen receptor; AR = androgen receptor; FDA = US Food and Drug Administration; EXTEND = Extended Tasks on Endocrine Disruption; WoE, weight of evidence.

*Source:* Modified from Burden et al. (2022).

**TABLE 2:** An overview of the Organisation for Economic Co‐operation and Development adverse outcome pathways on vertebrate endocrine disruption

OECD project	Status	ED modality	AOP title	Type of NAM deployed

1.12	EAGMST under review	Estrogen	Estrogen receptor antagonism leading to reproductive dysfunction	In vitro, in vivo
1.73	Under development	Estrogen	Binding to ER-a in immune cells leading to exacerbation of systemic lupus erythematosus	In vitro, in vivo
1.79	Under development	Estrogen	Early-life stromal ER activation by ED chemicals in the mammary gland leading to enhanced cancer risk	Not described
1.12	TFHA/WNT endorsed	Steroidogenesis	Aromatase inhibition leading to reproductive dysfunction	In vitro, in vivo
1.21	EAGMST under review	Steroidogenesis	Aromatase (Cyp19a1) reduction leading to impaired fertility in adult female	In vitro, in vivo
1.9	Under development	Steroidogenesis	5a-reductase inhibition leading to short anogenital distance in male (mammalian) offspring	In vitro, in vivo
1.9	Under development	Steroidogenesis	Decreased testosterone synthesis leading to short anogenital distance in male (mammalian) offspring	In vitro, in vivo
1.12	TFHA/WNT endorsed	Androgen	Androgen receptor agonism leading to reproductive dysfunction (in repeat-spawning fish)	In vitro, in vivo
1.26	Under development	Androgen	Androgen receptor activation leading to hepatocellular adenomas and carcinomas (in mouse and rat)	In vitro
1.9	Under development	Androgen	Androgen receptor antagonism leading to short anogenital distance in male (mammalian) offspring	In vitro
1.1	TFHA/WNT endorsed	Thyroid	Inhibition of thyroperoxidase and subsequent adverse neurodevelopmental outcomes in mammals	In vitro, in vivo
1.35	EAGMST under review	Thyroid	Deiodinase 1 inhibition leading to increased mortality via reduced anterior swim bladder inflation	In vitro, in vivo
1.35	EAGMST under review	Thyroid	Deiodinase 1 inhibition leading to increased mortality via reduced posterior swim bladder inflation	In vitro, in vivo
1.35	EAGMST under review	Thyroid	Deiodinase 2 inhibition leading to increased mortality via reduced anterior swim bladder inflation	In vitro, in vivo
1.35	EAGMST under review	Thyroid	Deiodinase 2 inhibition leading to increased mortality via reduced posterior swim bladder inflation	In vitro, in vivo
1.35	EAGMST under review	Thyroid	Thyroperoxidase inhibition leading to increased mortality via reduced anterior swim bladder inflation	In vitro, in vivo
1.59	Under development	Thyroid	Inhibition of thyroid peroxidase leading to impaired fertility in fish	In vitro, in vivo
1.9	Under development	Thyroid	Upregulation of thyroid hormone catabolism via activation of hepatic nuclear receptors and subsequent adverse neurodevelopmental outcomes in mammals	Not described
1.4	Under development	Thyroid	Kidney dysfunction by decreased thyroid hormone	In vitro, in vivo
1.84	Under development	Thyroid	Thyroid receptor antagonism and subsequent adverse neurodevelopmental outcomes in mammals	In vivo

OECD = Organisation for Economic Co-operation and Development; ED = endocrine disruption/disruptor; AOP = adverse outcome pathway; EAGMST = Extended Advisory Group on Molecular Screening and Tox-icogenomics; ER = estrogen receptor; TFHA = Task Force on Hazard Assessment; WNT = Working Group of the National Coordinators for the Test Guidelines Programme; Cyp19a1 = cytochrome P450 19a1.

## Data Availability

There is no data generated in this review.
