# Supplementary material for: New Approach Methodologies for the Endocrine Activity Toolbox: Environmental Assessment for Fish and Amphibians
Source: Environ Toxicol Chem. Author manuscript; Available in PMC 2024 Apr 1. (PMC10258674; doi:10.1002/etc.5584)
Supplement: Supplement1 [file NIHMS1894463-supplement-Supplement1.docx]

**Table of Contents**

| **Assay** | **Page** |
| --- | --- |
| EASZY Assay: Detection of Endocrine Active Substance, acting through estrogen receptors, using transgenic cyp19a1b-GFP Zebrafish embryos | 2-3 |
| RADAR Assay: Rapid Androgen Disruption Adverse-outcome Reporter Assay | 4-6 |
| REACTIV Assay: Rapid Estrogen Activity Tests In Vivo | 7-8 |
| XETA: Xenopus Eleutheroembryonic Thyroid Assay | 9-11 |
| YES: Saccharomyces cerevisiae Yeast Estrogen Screen | 12-13 |
| A-YES: Arxula adeninivorans Yeast Estrogen Screen | 14-15 |
| Performance-Based Test Guideline for Stably Transfected Transactivation In Vitro Assays to Detect Estrogen Receptor Agonists and Antagonists | 16-18 |
| H295R Steroidogenesis Assay | 19-21 |
| Androgen Receptor TransActivation Assays for Detection of Androgenic Agonist and Antagonist Activity of Chemicals using Stably Transfected Cell Lines | 22-24 |
| Performance-Based Test Guideline for Human Recombinant Estrogen Receptor (hrER) In Vitro Assays to Detect Chemicals with ER Binding Affinity | 25-26 |
| Androgen Receptor Binding (Rat Prostate Cytosol) | 27-28 |
| Aromatase (Human Recombinant) | 29-30 |
| Estrogen Receptor Binding Assay Using Rat Uterine Cytosol (ER-RUC) | 31-32 |
| Estrogen Receptor Transcriptional Activation (Human Cell Line HeLa-9903) Assay | 33-34 |
| Steroidogenesis (Human Cell Line – H295R) Assay | 35-37 |
| Human Sodium/Iodide Symporter (NIS) Inhibition Radioactive Iodide Uptake (RAIU) Assay | 38-39 |
| Human Iodothyronine Deiodinase (DIO1) inhibition assay, colorimetric iodide | 40-42 |
| Human Iodothyronine Deiodinase (DIO2) inhibition assay, colorimetric iodide | 43-45 |
| Human Iodothyronine Deiodinase (DIO3) inhibition assay, colorimetric iodide | 46-48 |
| Human Iodotyrosine Deiodinase (IYD) inhibition assay, colorimetric iodide | 49-51 |
| Amplex UltraRed Thyroperoxidase Inhibition Assay | 52-53 |

**EASZY Assay: Detection of Endocrine Active Substance, acting through estrogen receptors, using transgenic cyp19a1b-GFP Zebrafish embryos**

| **Category** | **Description** |
| --- | --- |
| Test Guideline(s) | [OECD 250](https://www.oecd.org/chemicalsafety/test-no-250-easzy-assay-detection-of-endocrine-active-substances-acting-through-estrogen-receptors-using-transgenic-tg-cyp19a1b-0a39b48b-en.htm) |
| Adopted/published | 17 June 2021 |
| Test species | *Danio rerio* (transgenic cyp19a1b-GFP zebrafish embryos) |
| Description | The EASZY assay is a mechanism-based screening assay (96h exposure) designed to detect endocrine active substance acting through estrogen receptors (ERs), to induce the expression of the green fluorescent protein (GFP) driven by the cyp19a1b promoter. |
| Endpoint(s) | Induction of fluorescence in eleuthero embryo’s radial glial cells. |
| Statistical endpoint summary | Should be aligned with other level-3 (embryo) assays. |
| Statistics | Hill linear regression model. |
| Endocrine Pathway(s) addressed | Estrogen |
| Validation status | Validation report summarizing a two phases validation exercise has been submitted to the OECD. |
| Validation approach | A two-phases validation study was carried out. Phase 1 demonstrated the transferability of the methods and addressed the repeatability and reproducibility of the assay within and between laboratories by testing 7 chemicals. An additional set of 16 single chemicals were tested in OECD intercalibration phase 1 and 2 by 4 laboratories to gain further information on the capacity of the assay to produce reliable and accurate data about the estrogenic activity of tested substances. |
| Domain(s) of applicability considerations | The test can be applied to substance types that have amenable physicochemical properties allowing for aquatic delivery. Bulky chemicals that cannot reach their molecular target in the brain because of their inability to pass through the brain-blood barrier could lead to a negative result (molecular weight >3kDa). The test is not suitable to test volatile chemicals. During the validation of the EASZY assay, only single chemicals were used and the applicability of the EASZY assay for evaluating binary or multi-component mixtures was not addressed. |
| Strengths | - OECD CF level 3 assay - Medium Throughput - 4 days (96 hours) - Test does not use protected life stages of animals - Quantitative readout |
| Weaknesses/Limitations | - Pharmacodynamics might be different between fertilized eggs and newly hatched embryos. - Hatching success potentially interferes with endocrine responses. - There is limited knowledge metabolic activity at this life stage. - The estrogen induced-GFP can be inhibited by substances that negatively interfere with the ER-signaling pathway (for instance there is a negative cross-talk between TCDD-induced AhR signaling pathway with the estrogen-induced cyp19a1b expression). If such negative interactions are suspected, EASZY may not be appropriate to report data for regulatory purpose. - Facilities to carry out experiments with transgenic organisms are needed - Not suitable for volatile chemicals. |
| Opportunities | This assays can be combined with a FET (OCDE TG 236) realizing FET on Cyp19a1b transgenic line can help in the concentration range finding and give inputs on the necessity to run a complete EASZY assay. |
| Concentration Setting | The maximum test concentration should be set by the solubility limit of the test chemical in the test medium, the maximum tolerated concentration (MTC), or a maximum concentration of 100 mg/L, whichever is lowest. If toxicity data is available on the test chemical, then expert judgement could be used to determine the maximum test concentration. If no relevant acute toxicity is available on the test chemical, a range-finding study should be performed on embryos to evaluate possible toxicity. At least three test concentrations should be used. They should be arranged in a geometric series with a separation factor not exceeding 10. Only one replicate with 10 embryos is prepared per test condition (test concentrations and control). |
| Test Acceptance criteria | - The fertilization rate of the eggs collected from the batches should be ≥70%. - Mortality on controls should not exceed 20% at the end of the test. - The mean measured fold induction of GFP induced by the reference substance should be ≥9 as compared to controls. |
| Animal Minimization | Non-protected animals are used. However, large numbers of non-protected life stages are used. If 2 runs give reproducible results (e.g., both runs indicate the tested chemical as positive) it is not necessary to conduct a third run. If not, or if a higher degree of certainty is required regarding the outcome of the assay, at least three independent runs should be conducted. |
| Animal Numbers | 20 embryos per conditions/ a minimum of 3 concentrations by chemical in addition to controls. |
| Solvent delivery | Solvents can be used as appropriate for the test substances. |
| Species Effectiveness | Zebrafish Fish Tg: Cyp19a1b-GFP |
| Connection to existing regulatory frameworks | As EASZY was still under validation when the OECD GD 150 was updated, EASZY is listed in this Guidance Document as a “Non-OECD non-mammalian screens and tests – Conceptual framework level 3”. EASZY is mentioned in the EFSA-ECHA GD for EDs identification. However as during the drafting of the document the test was not validated yet, no details are given on how to integrate EASZY in an ED testing strategy. |
| Commercial availability | The line can be purchase from INERIS |

**RADAR Assay: Rapid Androgen Disruption Adverse-outcome Reporter Assay**

| **Category** | **Description** |
| --- | --- |
| Test Guideline(s) | [OECD 251](https://www.oecd.org/publications/test-no-251-rapid-androgen-disruption-activity-reporter-radar-assay-da264d82-en.htm) |
| Adopted/published | 30 June 2022 |
| Test species | *Oryzias latipes* (transgenic with the stickleback Spiggin1-GFP genetic construct) |
| Description | Medium throughput 96-hour screening assay to measure the response of eleutheroembryos potential of androgen active chemicals. Potential modulation of the androgen axis is measured using this gene as a biomarker since Spiggin1 expression is controlled by androgen axis signaling and is required for male reproductive behavior in stickleback in the mating season. The promoter is coupled to a reporter gene for GFP which can be measured by fluorescence. |
| Endpoint(s) | Induction of fluorescence in eleutheroembryos. |
| Statistical endpoint summary | Used for screening for higher tier tests. |
| Statistics | One tailed hypothesis testing (Dunns test or Dunnetts test) |
| Endocrine Pathway(s) addressed | Androgen (various mechanisms): AR agonists / antagonists, altered expression or activity of downstream enzymes in steroidogenesis such as aromatase and 5α-reductase. Potentially also modulators of sex steroid transport via interaction with sex steroid plasma binding proteins, altered cellular uptake and modulators of androgen clearance. |
| Validation status | The test has been validated in five laboratories with eight test chemicals, in addition to 17α-methyl testosterone and flutamide concentration ranges which were included in every experiment performed in the validation exercise. However, one of the five laboratories only tested five of the eight chemicals. |
| Validation approach | The validation focused on demonstrating the relevance of the assay (i.e., its ability to detect compounds that act on the androgen system via a range of modes of action). It also showed that the assay protocol is optimized for performance across laboratories. Expected inactive chemicals were also included to challenge the protocol, assay validation criteria and the method of statistical analysis. |
| Domain(s) of applicability | The test can be applied to substance types that have amenable physicochemical properties allowing for aquatic delivery with 24-hour renewal of solutions. It will likely be challenging for poorly soluble, volatile and unstable test substances. |
| Strengths | - OECD CF level 3 assay - Medium Throughput - 4 days (96 hours) - Test does not use protected life stages of animals - Lower cost than protected life stages - Specific to androgen axis - Quantitative readout |
| Weaknesses/ Limitations | - Use of solvent (DMSO 0.2%) in both the control and exposed groups - Extent of metabolism unknown - Facilities to carry out experiments with transgenic organisms are needed - Substances that emit fluorescence between 500 - 550 nm when excited at wavelengths of 450 - 500 nm that might accumulate in the eleutheroembryo. - Not suitable for volatile chemicals. - Currently no validation to support detection of modulators of sex steroid transport or excretion |
| Opportunities | Could be combined with mammalian data for IATA (see EFSA proposal) |
| Concentration Setting | The maximum test concentration should be set by the solubility limit of the test chemical in the test medium, the maximum tolerated concentration (MTC), or a maximum concentration of 100 mg/L, whichever is lowest. If toxicity data is available on the test chemical, then expert judgement could be used to determine the maximum test concentration. If no relevant acute toxicity is available on the test chemical, a range-finding study should be performed on embryos to evaluate possible toxicity. At least five concentrations should be used in addition to controls. |
| Test Acceptance criteria | For the test to be valid, the following criteria should be met for each run, and if they  are not, the run is considered invalid:   - A statistically significant induction of fluorescence should be measured between the solvent control group and the positive control. - The combined mortality and/ or malformations and invalid data due to poorly positioned eleutheroembryos should not exceed 10% in each control group and in at least five treatment groups.   Groups not meeting these criteria are considered compromised.  For the test to be valid, the following criteria should be met for the pool of the three runs,  and if they are not, all three runs are considered invalid:   - The mean fluorescence of the 17MT 10 µg/L control group should be at least 10% higher than the mean of fluorescence of the 17MT 3 µg/L control group. - A statistically significant inhibition of fluorescence should be measured between the 17MT 3 µg/L control group and the 17MT 3 µg/L + flutamide 500 µg/L control group. - For the pool of the three runs, a test should have at least five uncompromised test concentrations. |
| Animal Minimization | Non-protected animals are used. However, large numbers of non-protected life stages are used. |
| Animal Numbers | With five test concentrations and the non-optional controls including either a test medium control or solvent control, performed in three runs, the RADAR assay uses 280 eleutheroembryos per run, therefore, 840 eleutheroembryos are required for all three runs constituting an experiment. |
| Solvent delivery | Solvent use is required for the stock solutions of the controls, and therefore included in the exposed groups at the same concentration (*i.e*., DMSO 0.2%) |
| Species Effectiveness | *Oryzias latipes* Spg1-GFP only |
| Connection to existing regulatory frameworks | The RADAR assay is intended to be a screening tool classifying the chemicals into potentially androgen active or inactiveThe RADAR assay is a level 3 test of the OECD conceptual framework for the testing of endocrine disrupters. Described in OECD GD150 (OECD, 2018a). Described in the ECHA/EFSA GD 2018. |
| Commercial availability of the assays | The spg1-gfp line will be accessible to laboratories from OECD member countries through WatchFrog as well as through partner laboratories with standard Fair Reasonable and Non-Discriminatory (FRAND) agreements compliant with OECD requirements. It is envisaged that these partner laboratories will form a network of distributors. The access model being followed will be the same as that for the *Xenopus* Embryonic Thyroid Assay (XETA) which has recently been adopted by the OECD (TG 248). |

**REACTIV Assay: Rapid Estrogen Activity Tests In Vivo**

| **Category** | **Description** |
| --- | --- |
| Test Guideline(s) | Currently under OECD validation |
| Adopted/published | Expected 2023 |
| Test species | *Oryzias latipes* (transgenic with the ChgH-GFP genetic construct) |
| Description | Medium throughput 24-hour screening assay to measure the response of eleutheroembryos to potential estrogen axis active chemicals. Potential modulation of the estrogen axis is measured by the use of this gene as biomarker the Choriogenin H gene is controlled by estrogen axis signaling and is required for egg production in the mating season. The promoter is coupled to a reporter gene for GFP which can be measured by fluorescence. |
| Endpoint(s) | Induction of fluorescence in eleutheroembryos. |
| Statistical endpoint summary | Used for screening for higher tier tests. |
| Statistics | One tailed hypothesis testing (Dunns test or Dunnetts test) |
| Endocrine Pathway(s) addressed | Estrogen |
| Validation status | Ongoing publication of the OECD validation report is expected in 2023. |
| Validation approach | The validation will focus on demonstrating the relevance of the assay (i.e., its ability to detect compounds that act on the estrogen system via a range of modes of action.) It is also expected to show that the assay protocol is optimized for performance across laboratories. Expected inert chemicals will also be included to challenge the protocol, assay validation criteria and the method of statistical analysis. |
| Domain(s) of applicability | This test can be applied to substance types that have amenable physicochemical properties allowing for aquatic delivery with 24 hour renewal of solutions (will be challenging for poorly soluble, volatile and instable test substances). |
| Strengths | - Under validation as a non-animal OECD CF level 3 assay - Medium Throughput - 1 day (24 hours) - Test does not use protected life stages of animals - Specific to estrogen axis - Quantitative readout |
| Weaknesses/Limitations | - Extent of metabolism unknown - Facilities to carry out experiments with transgenic organisms are needed - Substances that emit fluorescence between 500 - 550 nm when excited at wavelengths of 450 - 500 nm that might accumulate in the eleutheroembryo. - Not suitable for volatile chemicals. - Currently no validation to support detection of modulators of sex steroid transport or excretion |
| Opportunities | Could be combined with mammalian data for IATA (see EFSA proposal) |
| Concentration Setting | The maximum test concentration should be set by the solubility limit of the test chemical in the test medium, the maximum tolerated concentration (MTC), or a maximum concentration of 100 mg/L, whichever is lowest. If toxicity data is available on the test chemical, then expert judgement could be used to determine the maximum test concentration. If no relevant acute toxicity is available on the test chemical, a range-finding study should be performed on embryos to evaluate possible toxicity. |
| Test Acceptance criteria | Validity criteria will be finalized following the OECD interlaboratory validation exercise. |
| Animal Minimization | No protected animals are used. However, large numbers of non-protected life stages are used. |
| Animal Numbers | No protected animals are used. Exact numbers of eleuthero-embryos will be determined and agreed during the initial phases of the OECD validation. |
| Solvent delivery | Solvent use is required for the stock solutions of the controls, and therefore included in the exposed groups at the same concentration (*i.e*., DMSO 0.2%). |
| Species Effectiveness | *Oryzias latipes* ChgH-GFP only |
| Connection to existing regulatory frameworks | The REACTIV assay is intended to be a screening tool classifying the chemicals into potentially estrogen active or inactive. The REACTIV assay is under validation at level 3 of the OECD conceptual framework for the testing of endocrine disrupters. |
| Commercial availability of the assays | The chgh-gfp line will be accessible to laboratories from OECD member countries through WatchFrog as well as through partner laboratories with standard Fair Reasonable and Non-Discriminatory (FRAND) agreements compliant with OECD requirements. It is envisaged that these partner laboratories will form a network of distributors. The access model being followed will be the same as that for the *Xenopus* Embryonic Thyroid Assay (XETA) which has recently been adopted by the OECD (TG 248). |

**XETA: Xenopus Eleutheroembryonic Thyroid Assay**

| **Category** | **Description** |
| --- | --- |
| Test Guideline(s) | [OECD TG 248](https://www.oecd-ilibrary.org/environment/tg-248-xenopus-eleutheroembryonic-thyroid-assay-xeta_a13f80ee-en) |
| Adopted/published | June 2019 |
| Test species | *Xenopus laevis* (transgenic with the THb/ZIP-GFP genetic construct) |
| Description | Medium throughput 72-hour screening assay to measure the response of eleuthero embryos to potentially thyroid active chemicals using transgenic *X.* *laevis* embryos. Potential modulation of thyroid is measured by the use of this thyroid-response gene as biomarker since the TH/bZIP expression triggers metamorphosis and, in-part, controls its timing. The promoter is coupled to a reporter gene for GFP which can be measured in whole eleutheroembryos by fluorescence. |
| Endpoint(s) | Induction of fluorescence in eleutheroembryos. |
| Statistical endpoint summary | Statistically significant difference above 12% as compared to negative or positive control for at least one test concentration and including the highest test concentration (the 12% threshold was determined from the power analysis which found the power to exceed 80% for all concentration-response shapes simulated.) |
| Statistics | One tailed hypothesis testing (Williams’ test or Dunnett’s test) |
| Endocrine Pathway(s) addressed | Thyroid |
| Validation status | OECD has published a validation report summarizing a two-phase validation exercise leading to the publication of the Test Guideline. |
| Validation approach | The phase I validation focused on demonstrating the relevance of the assay, (i.e., its ability to detect compounds that act on the thyroid system.) Phase I also showed that the assay protocol is optimized for performance across laboratories. The phase II validation included a demonstration of the benefits of the efficiency and medium-throughput-nature of this test in screening for potential thyroid active compounds. Phase II examined the ability of the protocol to test a wider range of thyroid active chemicals with different modes of action (expanding the modes that were tested in phase I) and additional negative substances to challenge the protocol and the statistical method of analysis. |

| Domain(s) of applicability | This test can be applied to substance types that have amenable physicochemical properties allowing for aquatic delivery with 24 hour renewal of solutions (will be challenging for poorly soluble, volatile and instable test substances). |
| --- | --- |
| Strengths | - OECD CF level 3 assay - Medium Throughput - 3 days (72 hours) - Test does not use protected life stages of animals - Quantitative readout |
| Weaknesses/Limitations | - Not validated for all thyroid mechanisms - Extent of metabolism not fully characterized - Facilities to manage transgenic organisms - Response range of fluorescence data small - Substances that emit fluorescence between 500 - 550 nm when excited at wavelengths of 450 - 500 nm that might accumulate in the eleutheroembryo not suitable. - Not suitable for volatile chemicals. - TH synthesis inhibitors not sufficiently included in the validation, but some were shown to be detected in publications. |
| Opportunities | This assay could be combined with mammalian data to for IATA (see EFSA proposal). |
| Concentration Setting | The maximum test concentration should be set by the solubility limit of the test chemical in the test medium, the maximum tolerated concentration (MTC), or a maximum concentration of 100 mg/L, whichever is lowest. If toxicity data is available on the test chemical, then expert judgement could be used to determine the maximum test concentration. If no relevant acute toxicity is available on the test chemical, a range-finding study should be performed on embryos to evaluate possible toxicity. |
| Test Acceptance criteria | The following criteria should be met for each run and for the pool of the three runs to confirm the validity of the test:   - A statistically significant induction of fluorescence should be measured between the test medium control group and the T3 control group (at least 20% higher). - A statistically significant induction of fluorescence of at least 70% should be present between the T4 control group and the test medium control. - The coefficient of variation of the fluorescence intensity measured for the test medium control should not exceed 30%. - The initial pH of the exposure solutions should be between 6.5 and 8.5 for each renewal. - Mortality and percentage of malformed organisms should not each exceed 10% in each control group. - Proficiency chemicals – T4, propylthiouracil, abamectin, methomyl. |
| Animal Minimization | Non-protected animals are used. However, large numbers of non-protected life stages are used. |
| Animal Numbers | 540 – 3 test treatments without T3, 3 test treatments with T3, 1 test medium control, T3 control, and T4 control with 20 organisms per treatment and performed 3 times (runs). Solvent group (if necessary) would require an additional 120 embryos |
| Solvent delivery | No specific guidance is given beyond reference to OECD GD 23. Note some approaches may not be applicable due to the reduced format of the test system. |
| Species Effectiveness | *Xenopus laevis* TH/bZIP |
| Connection to existing regulatory frameworks | The XETA is intended to be an amphibian screen classifying the chemicals into potentially thyroid active or inactive. The XETA is placed at level 3 of the OECD conceptual framework for the testing of endocrine disrupters (OECD, 2018). The OECD GD 150 provides further guidance on the interpretation and extrapolation between taxa of the results of the XETA (OECD, 2018a). Described in the ECHA/EFSA GD 2018 – however, at the time it was not a validated assay. EFSA has since released some guidance on how the XETA may be used in the context of the ECHA/EFSA GD 2018. |
| Commercial availability of the assays | The THbZIP-GFP *Xenopus* is maintained in the lead lab (Watchfrog) and in different OECD countries in 3 public laboratories located in USA (University of Cincinnati), Portugal (University of Aveiro) and France (Museum of Natural History) and one CRO located in Japan (IDEA consulting). |

**YES: *Saccharomyces cerevisiae* Yeast Estrogen Screen**

| **Category** | **Description** |
| --- | --- |
| Test Guideline(s) | [ISO 19040-1:2018](https://www.iso.org/standard/64450.html) |
| Adopted/published | August 2018 |
| Test species | *Saccharomyces cerevisiae* (Guidance covers two different strains with different validity criteria. The main one is described here; the other is mentioned in appendix G of the ISO 19040-1 document) |
| Description | Reporter gene assay in the yeast *S. cerevisiae.* Used for measurement of activation of human estrogen receptor (hERα). Activation is measured by induction of the reporter gene which encodes β-galactosidase. The activity of β-galactosidase as a measure for estrogenic activity is determined by using a substrate for the enzyme; chlorophenol red-β-D-galactopyranoside (CPRG). β-Galactosidase converts the yellow-orange CPRG substrate into the red chromophore chlorophenol red, which can be measured photometrically. |
| Endpoint(s) | Photometric detection of chlorophenol red. |
| Statistical endpoint summary | Calculation of 17β-estradiol equivalents (EEQ) of valid sample dilutions from E2 standard curve |
| Statistics | The EC_50_ of the standard curve should be determined by an appropriate statistical method, such as probit analysis, moving average or binomial methods |
| Endocrine Pathway(s) addressed | Estrogenic pathway |
| Validation status | Validated in 2016, data published in 2017 |
| Validation approach | Interlaboratory test with 16 laboratories. 8 samples were tested, 6 aqueous and 2 ethanolic.  Effluent of municipal sewage treatment plant (spiked and un-spiked), influent to a municipal STP, surface water (spiked and un-spiked), mixture of chemicals. All laboratories tested the aqueous samples, 10 did the ethanolic samples. Two to three repeated measures were performed per sample. |
| Domain(s) of applicability | The test can be applied to the following categories (but it will likely be challenging for poorly soluble, volatile and unstable test substances): Fresh water, waste water, aqueous extracts and leachates, eluates of sediments (fresh water), pore water, aqueous solutions of single substances or of chemical mixtures, drinking water. The yeast cells tolerate an ethanol concentration of 0.2%. Extracts and compounds prepared in organic solvents can be used. Then the solvent is evaporated off before reconstituting with water and inoculating with yeast. |
| Strengths | - No animals used - No possibility for cross talk between other hormonal pathways – estrogen specific - Can provide mechanistic information |
| Weaknesses/Limitations | - Since in cells, results should not be directly extrapolated to the complex signaling and regulation of the intact endocrine system *in vivo* - Cytotoxicity of samples can mask estrogenic effects - Laboratories might need approval for work with genetically modified organisms |
| Opportunities | This assay could be used for screening for endocrine activity and to prioritize compounds. |
| Concentration Setting | The limit of quantification (LOQ) of this method for the direct analysis of water samples is between 8 ng/l and 15 ng/l 17β-estradiol equivalents (EEQ) based on the results of the international interlaboratory trial. The upper threshold of the dynamic range for this test is between 120 ng/l and 160 ng/l 17β-estradiol equivalents (EEQ). Samples showing estrogenic potencies above this threshold have to be diluted for a valid quantification. Extraction and pre-concentration of water samples can prove necessary, if their estrogenic potential is below the given LOQ. |
| Test Acceptance criteria | EC_50_ of E2 should be between 39 ng/L and 107 ng/L. Mean corrected absorbance for negative control should be in the range of 0.05-1.5 The relative std of corrected absorbance for the negative control should be lower than 30%. Induction rate by the highest E2 concentration should be larger than 10. A dilution level of a sample is valid if the relative growth rate of the cells is >0.7 x <1.3 and the inter well deviation is < 15% |
| Animal Minimization | No animals used. |
| Animal Numbers | 0 |
| Solvent delivery | Both aqueous solutions, extracts and organic solvents can be used to prepare the test |
| Species Effectiveness | Human estrogen receptor alpha |
| Connection to existing regulatory frameworks | The assay is not included in part A or B in OECD 150. Listed in the OECD Conceptual Framework for Testing and Assessment of Endocrine Disrupting Chemicals, and in table 9 referring to the OECD CF in the ECHA/EFA guidance. |
| Commercial availability | Test kits are available for purchase. |

**A-YES: *Arxula adeninivorans* Yeast Estrogen Screen**

| **Category** | **Description** |
| --- | --- |
| Test Guideline(s) | [ISO 19040-2:2018](https://www.iso.org/standard/64451.html) |
| Adopted/published | August 2018 |
| Test species | *Arxula adeninivorans* |
| Description | Reporter gene assay used for measurement of the activation of human estrogen receptor alpha (hERα). Activation of the receptor is measured by induction of the reporter gene phyK which encodes phytase. The activity of phytase as a measure for estrogenic activity is determined by using a substrate for phytase (4-nitrophenylphosphate) which is cleaved to a colored product (4-nitrophenol) that can be measured photometrically. |
| Endpoint(s) | Photometric determination of 4-nitrophenol. |
| Statistical endpoint summary | Calculation of EC_50_ in terms of EEQ equivalents. |
| Statistics | The EC_50_ of the standard curve should be determined by an appropriate statistical method, such as probit analysis, moving average or binomial methods |
| Endocrine Pathway(s) addressed | Estrogenic pathway. |
| Validation status | Interlaboratory trial carried out in 2015 |
| Validation approach | 14 laboratories, 13 reported valid results. 9 water samples, including wastewater samples and surface water with and without EE2 spiking, field blank, mixture of single substances and saline water spiked with E2 and EE2. |
| Domain(s) of applicability | The test can be applied to the following categories (but it will likely be challenging for poorly soluble, volatile and unstable test substances): Fresh water, waste water, aqueous extracts and leachates, eluates of sediments (fresh water), pore water, aqueous solutions of single substances or of chemical mixtures, drinking water. |
| Strengths | - No animals used - No possibility for cross talk between other hormonal pathways – estrogen specific - Can provide mechanistic information - Kits are commercially available |
| Weaknesses/Limitations | - Since in cells, results should not be directly extrapolated to the complex signaling and regulation of the intact endocrine system *in vivo* - Potential for masking of estrogenic effects by matrix effect, most relevant for environmental water samples, less for aqueous solutions of substances/mixtures - Cytotoxicity of samples can mask estrogenic effects - Laboratories might need approval for work with genetically modified organisms |
| Opportunities | This assay could be used for screening for endocrine activity and to prioritize compounds. |
| Concentration Setting | The limit of quantification (LOQ) of this method for the direct analysis of water samples is between 1,5 ng/l and 3 ng/l 17β-estradiol equivalents (EEQ). The upper threshold of the dynamic range for this test is between 25 ng/l and 40 ng/l 17β-estradiol equivalents (EEQ). Samples showing estrogenic potencies above this threshold have to be diluted for a valid quantification. Extraction and pre-concentration of water samples can prove necessary, if their estrogenic potential is below the given LOQ. |
| Test Acceptance criteria | EC_50_ of E2 should be between 7 ng/L and 35 ng/L. The critical concentration (above this an estrogenic effect is measurable) is ≤ 8 ng/L E2. The ratio of the curve plateau divided by bottom curve point is ≥4 and ≤ 20. The ratio between mean corrected absorbance at 405 nm of replicates for 80 ng/L E2 and the curve plateau is ≥ 0.75. The mean relative standard deviation of corrected absorbance at 405 nm for replicates for the negative control and the dilution series of E2 is ≤ 12%. A dilution level of a sample is valid if the relative growth of yeast cells in this dilution is ≥0.3 x ≤3.5 and the relative standard deviation of the corrected absorbance at 405 nm of replicates is ≤ 15%. |
| Animal Minimization | No animals needed |
| Animal Numbers | 0 |
| Species Effectiveness | Human estrogen receptor alpha |
| Connection to existing regulatory frameworks | The assay is not included in part A or B in OECD 150. Listed in the OECD Conceptual Framework for Testing and Assessment of Endocrine Disrupting Chemicals, and in table 9 referring to the OECD CF in the ECHA/EFA guidance. |
| Commercial availability | Test kits are available for purchase. |

**Performance-Based Test Guideline for Stably Transfected Transactivation In Vitro Assays to Detect Estrogen Receptor Agonists and Antagonists**

| **Category** | **Description** |
| --- | --- |
| Test Guideline(s) | [OECD TG 455](https://www.oecd.org/env/test-no-455-performance-based-test-guideline-for-stably-transfected-transactivation-in-vitro-assays-to-detect-estrogen-receptor-9789264265295-en.htm) |
| Adopted/published | September 2016 |
| Test species | Human (h) ERα-HeLa-9903 and VM7Luc4E2 cell line |
| Overview of assay | Estrogen receptor agonists and antagonists can be determined by exposing the cells to test substances. Changes in luciferase compared to controls will show estrogenic receptor activation or inhibition depending on the direction. |
| Endpoint(s) | Luciferase |
| Statistics | Both qualitative (e.g., positive/negative) and/or quantitative (e.g., EC_50_) assessments of ER-mediated activity should be based on empirical data and sound scientific judgment. Where possible, positive results should be characterized by both the magnitude of the effect as compared to the vehicle (solvent) control or reference estrogen and the concentration at which the effect occurs. |
| Endocrine Pathway(s) addressed | Estrogen |
| Validation status | Validation studies of the assay have demonstrated their relevance and reliability for their intended purpose |
| Validation approach | Using known estrogen agonists and antagonists |
| Domain(s) of applicability | Substances that are water soluble or in their correct solvent can be tested in this assays.  Special considerations will need to be applied to those compounds that are highly volatile. In such cases, nearby control wells may generate false positives, and this should be considered in light of expected and historical control values. In the few cases where volatility may be of concern, the use of “plate sealers” may help to effectively isolate individual wells during testing and is therefore recommended in such cases. This test system cannot capture metabolic activation. |
| Strengths | - No animals - Quick (exposures are 20-24 hours) - Could be high-throughput - Can provide mechanistic information in WoE |
| Weaknesses/Limitations | - Since in cells, results should not be directly extrapolated to the complex signaling and regulation of the intact endocrine system *in vivo* - Other mechanisms through which ED can occur, including (i) interactions with other receptors and enzymatic systems within the endocrine system, (ii) hormone synthesis, (iii)metabolic activation and/or inactivation of hormones, (iv) distribution of hormones to target tissues, and (v) clearance of hormones from the body. None of the test methods under this PBTG addresses these modes of action. - Downstream interactions would not be measured by this assay - Some substances display both agonist and antagonist acidity (could be cell type dependent) – known as Selective estrogen receptor modulators (SERMs). Chemicals that are negative could be evaluated in an ER binding assays before concluding the substance does not bind the receptor. - Potential for chemicals to increase chemiluminescence via non-ER mechanisms, thus possibly giving a false positive response. |
| Opportunities | Could be used for screening |
| Concentration Setting | The concentrations tested should remain within the solubility range of the test chemicals and not demonstrate cytotoxicity. Initially, chemicals are tested up to the maximum concentration of 1 µL/mL, 1 mg/mL, or 1 mM, whichever is the lowest. Based on the extent of cytotoxicity or lack of solubility observed in the preliminary test, the first definite run should test the chemical at log-serial dilutions starting at the maximum acceptable concentration (e.g., 1 mM, 100µM, 10µM, etc.) and the presence of cloudiness or precipitate or cytotoxicity noted. Less than 80% cell viability is considered cytotoxic. |
| Test Acceptance criteria | - Data should be sufficient for a quantitative assessment of ER activation (for agonist assay) or suppression (for antagonist assay) (i.e., efficacy and potency). - The mean reporter activity for the reference concentration of reference estrogen should be at least the minimum specified in the test methods relative to that of the vehicle (solvent) control to ensure adequate sensitivity. For the STTA and VM7Luc ER TA test methods, this is four times that of the mean vehicle control on each plate. - The concentrations tested should remain within the solubility range of the test chemicals and not demonstrate cytotoxicity. - Consistent results should be achieved in at least two out of two or three runs of the assay. To be acceptable, the results should also meet the performance standards given in the assay. Small deviations are unlikely to have compromised the assay, but judgement should be made on a case-by-case basis. - Demonstration of laboratory proficiency with proficiency chemicals is required at the outset: 14 for the agonist assay and 10 for the antagonist assay. - The assay requires a minimum of 80% cell viability |
| Animal Minimization | No animals are used |
| Animal Numbers | 0 |
| Solvent delivery | Dependent on the substances tested. DMSO, water, and ethanol (95% to 100% purity) are suitable solvents. In case DMSO is used as solvent, the maximum concentration of DMSO during incubation should not exceed 1% (v/v). Prior to use, the solvent should be tested for absence of cytotoxicity and interference with the assay performance |
| Species Effectiveness | Both cell lines are human derived. Could miss agonists and antagonists in other species because of differences in receptors |
| Connection to existing regulatory frameworks | Listed in “Revised Guidance Document 150 on Standardized Test Guidelines for Evaluating Chemicals for Endocrine Disruption. |
| Commercial availability of the assays | Proficiency substances and luciferase kits are commercially available. The stably transfected hERα-HeLa-9903 cell line should be used for the assay. The cell line can be obtained from the Japanese Collection of Research Bioresources (JCRB) Cell Bank2, upon signing a Material Transfer Agreement (MTA). |

**H295R Steroidogenesis Assay**

| **Category** | **Description** |
| --- | --- |
| Test Guideline(s) | [OECD TG 456](https://www.oecd.org/env/ehs/testing/Test%20No.456-English.pdf) |
| Adopted/published | July 2011 |
| Test species | Human (h) NCI-H295R cell line |
| Overview of assay | Inducers and inhibitors of the production of testosterone and 17β-estradiol are determined by exposing H295R cells to substances of interested. Changes in the production of each hormone are compared to the solvent controls and increases and decreases are reported as fold-changes relative to these controls. Cytotoxicity/cell viability is assessed in parallel in each well. |
| Endpoint(s) | Concentrations of testosterone and 17β-estradiol as determined by validated enzyme-linked immunoassays (ELISA) or LC-MS. |
| Statistics | Results should be normalized to the mean solvent control value of each test plate, and results expressed as changes relative to the control (solvent control) in each test plate. All data are to be expressed as mean ± 1 standard deviation (SD). Only data from treatment groups with greater or equal to 80% cell viability is to be considered for downstream analyses. Depending on the concentration-response profile data should be evaluated either using type 1 (i.e., parametric or non-parametric ANOVA followed by the appropriate post-hoc tests [Dunnett’s Test or Mann Whitney U Test] for incomplete concentration response curves where the two greatest concentrations are on the linear portion of the curve) or type 2 (regression models where a full concentration-response curve can be modeled) statistics. |
| Endocrine Pathway(s) addressed | Steroidogenesis |
| Validation status | Validation studies were conducted and completed under the guidance by the OECD NA VMG following an international ring-test format using 28 test compounds. During this validation study the relevance and reliability of the H295R Steroidogenesis Assay has been demonstrated its intended purpose. |
| Validation approach | Using known endocrine active compounds with diverse mechanisms of action including strong, medium and weak inducers of different steroidogenic processes as well as negative compounds. |
| Domain(s) of applicability | Substances that are water soluble or in their correct solvent can be tested in this assays. Special considerations will need to be applied to those compounds that are highly volatile. In such cases, nearby control wells may generate false positives, and this should be considered in light of expected and historical control values. In the few cases where volatility may be of concern, the use of “plate sealers” may help to effectively isolate individual wells during testing and is therefore recommended in such cases. This test system cannot capture metabolic activation. This test system only captures very limited metabolic activation that is described incompletely for this cell line. |
| Strengths | - No animals - Quick (exposures are 48 hours) - Could be high-throughput - Can provide mechanistic information in WoE - Integrative effect on hormone production capturing all processes along the steroidogenic pathway - Can be modified to target specific enzymes |
| Weaknesses/Limitations | - Since in cells, results should not be directly extrapolated to the complex signaling and regulation of the intact endocrine system *in vivo.* - Complex culture protocol requiring a certain cell age and passage window for commencing the assay. - Other mechanisms through which ED can occur, including (i) interactions with hormone receptors, (ii) metabolic activation and/or inactivation of hormones, (iii) distribution of hormones to target tissues, and (iv) clearance of hormones from the body. None of the test methods under this PBTG addresses these modes of action. - Some substances may interact with antibody-based (e.g. ELISA) hormone detection assays. All compounds to be tested have to be assessed for potential cross-reactivity. Does not apply to LC-MS hormone detection systems. - Hormone endpoints not specific to a certain KE such as individual enzymes involved with synthesis of testosterone or and 17b-estradiol but rather capture a net effect on one or multiple targets along the entire steroidogenesis pathway. However, endpoints can be customized to assess expression or activities of specific enzymes such as aromatase, 3bHSD, etc. - H295R cells change their hormone-producing properties as a function of age (freeze-thaw cycles and passages); therefore, cells have to be grown using a specific pattern and assessed for baseline performance prior to commencing an experiment. |
| Opportunities | Could be used for high-throughput screening |
| Concentration Setting | The concentrations tested should remain within the solubility range of the test chemicals and not demonstrate cytotoxicity. Commonly, the assay is conducted in two tiers, with tier one typically covering a concentration range between 0.001 – 1000 mM if no a prior information on chemical solubility, cytotoxicity and other properties is available, and with tier 2 bracketing the active concentrations identified during tier 1 using half -log concentration spacing. |
| Test Acceptance criteria | Before using the assay, a laboratory should demonstrate that it is capable of achieving and maintaining appropriate cell culture and test conditions required for the successful conduct of the assay. When analyzed with the hormone measurement assay, the average recovery rates (based on triplicate measures) for the spiked amounts of hormone should not deviate more than 30% from nominal concentrations. No substantial (≥30 % of basal hormone production of the respective hormone) cross-reactivity with any of the hormones produced by the cells should occur. A minimum of two independent runs of the assay should be conducted. If consistent results have been achieved, then no further replications are required. In cases where results were ambiguous in the first two runs a third experiment has to be conducted. The minimum cell viability required per well is 80%, and wells with lower viability should not be included in the final data analysis. |
| Animal Minimization | No animals are used. |
| Animal Numbers | 0 |
| Non-solvent delivery | DMSO (95% to 100% purity) has been the only solvent validated for use with the assay to date. In case DMSO is used as solvent, the maximum concentration during incubation should not exceed 1% (v/v). If other solvents are intended to be used, they should be tested for absence of cytotoxicity and interference with assay performance. |
| Species Effectiveness | The cell line is human derived. However, given the conservation of the steroidogenic pathway across vertebrates it is assumed that this assay is applicable to all vertebrate taxa including fish, amphibians, reptiles, birds, and mammals. |
| Connection to existing regulatory frameworks | Listed in “Revised Guidance Document 150 on Standardized Test Guidelines for Evaluating Chemicals for Endocrine Disruption. |
| Commercial availability of the assays | H295R cells are available from ATCC (NCI-H295R). Hormone analytical kits are available from multiple manufacturers; however, they performance needs to be validated before use with the assay. |

**Androgen Receptor TransActivation Assays for Detection of Androgenic Agonist and Antagonist Activity of Chemicals using Stably Transfected Cell Lines**

| **Category** | **Description** |
| --- | --- |
| Test Guideline(s) | [OECD TG 458](https://www.oecd.org/env/test-no-458-stably-transfected-human-androgen-receptor-transcriptional-activation-assay-for-detection-of-androgenic-agonist-9789264264366-en.htm) |
| Adopted/published | June 2020 |
| Test species | 1. AR-EcoScreen^TM^ - Chinese hamster ovarian cancer cells 2. AR-CALUX^®^ - Human osteo-sarcoma cells 3. ARTA - Human prostate carcinoma epithelial cells |
| Overview of assay | This guideline covers three different assays for detecting chemicals that activate or inhibit androgen receptor-regulated reporter genes:   1. AR-EcoScreen^TM^ 2. AR-CALUX^®^ 3. ARTA   Androgen receptor (AR) agonists and antagonists can be determined by exposing the cells to substances of interest. Binding to the AR will trigger transcriptional activation and inhibition of an androgen-regulated reporter gene (firefly luciferase). Changes in luciferase compared to controls will show androgenic receptor activation or inhibition depending on the direction. |
| Endpoint(s) | Luminescence determined through a luciferase (firefly; *Photinus pyralis*) assay |
| Statistics | In all three tests data are normalized to a positive control in the agonist and antagonist assays, and both qualitative (e.g., positive/negative) and/or quantitative (e.g. EC_50_) assessments of AR-mediated activity should be based on empirical data and sound scientific judgment. Where possible, positive results should be characterized by both the magnitude of the effect as compared to the vehicle (solvent) control or reference androgen/antiandrogen and the concentration at which the effect occurs). |
| Endocrine Pathway(s) addressed | Androgen |
| Validation status | Validation studies of the AR-EcoScreen^TM^, AR-CALUX^®^ and ARTA assays have demonstrated their relevance and reliability for their intended purpose |
| Validation approach | Using know androgen agonists and antagonists |
| Domain(s) of applicability | Substances that are water soluble or in their correct solvent. Special considerations will need to be applied to those compounds that are highly volatile. In such cases, nearby control wells may generate false positives, and this should be considered in light of expected and historical control values. In the few cases where volatility may be of concern, the use of “plate sealers” may help to effectively isolate individual wells during testing and is therefore recommended in such cases.  Test systems:   - Cannot capture metabolic activation - Have not been validated for the use with mixtures |
| Strengths | - No animals - Quick (exposures are 20-24 hours plus 24 hour pre-incubation of cells) - Development of high-throughput assays underway - Can provide mechanistic information in WoE |
| Weaknesses/Limitations | - Since in cells, results should not be directly extrapolated to the complex signaling and regulation of the intact endocrine system *in vivo* - Does not capture other mechanisms through which ED can occur, including (i) interactions with other receptors and enzymatic systems within the endocrine system, (ii) hormone synthesis, (iii) - metabolic activation and/or inactivation of hormones, (iv) distribution of hormones to target tissues, and (v) clearance of hormones from the body. None of the test methods under this PBTG addresses these modes of action. - Downstream interactions would not be measured by this assay - Potential for chemicals to increase chemiluminescence via non-AR mechanisms:   - Some (very little) cross talk can occur with the glucocorticoid receptor (AR-EcoScreen^TM^ and AR-CALUS®), thus possibly giving a false positive response. Specificity can be assessed in the antagonistic assay.   - Some chemicals may interfere directly with luminescence |
| Opportunities | Could be used for screening |
| Concentration Setting | The concentrations tested should remain within the solubility range of the test chemicals and not demonstrate cytotoxicity. |
| Test Acceptance criteria | - Data should be sufficient for a quantitative assessment of AR activation (for agonist assay) or suppression (for antagonist assay) (i.e. efficacy and potency). - The mean reporter activity for the reference concentration of reference androgen should be at least the minimum specified in the test methods relative to that of the vehicle (solvent) control to ensure adequate sensitivity. For the AR-EcoScreenTM, AR-CALUX® and ARTA test methods, this is >6.4-, >20- and ³13-times that of the mean vehicle control on each plate, respectively. - The concentrations tested should remain within the solubility range of the test chemicals and not demonstrate cytotoxicity. - Consistent results should be achieved in at least two out of two or three runs of the assay. To be acceptable, the results should also meet the performance standards given in the assay. Small deviations are unlikely to have compromised the assay, but judgement should be made on a case-by-case basis. - Demonstration of laboratory proficiency with proficiency chemicals is required at the outset: 8 for the agonist assay and 9 for the antagonist assay. - The assay also requires a minimum of 80% cell viability |
| Animal Minimization | No animals used. |
| Animal Numbers | 0 |
| Non-solvent delivery | Dependent on the substances tested. DMSO, water and ethanol (95% to 100% purity) are suitable solvents. The maximum concentration of DMSO during incubation should not exceed 0.1% (v/v). Prior to use, the solvent should be tested for absence of cytotoxicity and interference with the assays performance |
| Species Effectiveness | Two of the cell lines (AR-CALUX and ARTA) are human derived. The AR-EcoScreenTM originated from hamster ovarian cancer cells. Could miss agonists and antagonists in other species because of differences in receptors – could be more of an issue in species with duplicated genomes. |
| Connection to existing regulatory frameworks | Listed in OECD 150 |
| Commercial availability of the assays | Whole assays (AR-CALUX®) or proficiency substances and luciferase kits are commercially available.  The cell lines used in the AR-EcScreenTM and ARTA can be obtained from the Japanese Collection of Research Bioresources (JCRB) Cell Bank2 and the Korean Cell Bank and Korean MFDS, respectively, upon signing a Material Transfer Agreement (MTA). The cell line used in the ER-CALUX® can be obtained after signing a license agreement with BDS. |

**Performance-Based Test Guideline for Human Recombinant Estrogen Receptor (hrER) In Vitro Assays to Detect Chemicals with ER Binding Affinity**

| **Category** | **Description** |
| --- | --- |
| Test Guideline(s) | [OECD TG 493](https://www.oecd.org/env/test-no-493-performance-based-test-guideline-for-human-recombinant-estrogen-receptor-hrer-in-vitro-assays-to-detect-chemicals-9789264242623-en.htm) |
| Adopted/published | July 2015 |
| Test species | Human recombinant ERα |
| Overview of assay | The assay measures the ability of a radiolabeled ligand ([3H]17βestradiol) to bind with the ER in the presence of increasing concentrations of a test chemical (i.e.  competitor). Test chemicals that possess a high affinity for the ER compete with the radiolabeled ligand at a lower concentration as compared with those chemicals with lower affinity for the receptor. The assay consists of two major components: a saturation binding experiment to characterize receptor-ligand interaction parameters and document ER specificity, followed by a competitive binding experiment that characterizes the competition between a test chemical and a radiolabeled ligand for binding to the ER. The performance-based test guideline (PBTG) was developed based on two reference test methods (Freyberher-Wilson and CERI). |
| Endpoint(s) | Binding affinity (IC_50_) [positive, negative, or equivocal result for ability to bind to ER] |
| Statistics | There is currently no universally agreed method for interpreting ER binding data. However, both qualitative (e.g. binder/non-binder) and/or quantitative (e.g. log IC_50_, Relative Binding Affinity (RBA), etc.) assessments of hrER-mediated activity should be based on empirical data and sound scientific judgment. Discussed in additional detail in TG paragraph 21. |
| Endocrine Pathway(s) addressed | Estrogen |
| Validation status | Validation studies of the CERI and FW binding assays have demonstrated their relevance and reliability for their intended purpose |
| Validation approach | Using known estrogen agonists and antagonists |
| Domain(s) of applicability | Not tested with mixtures (single components only)  Screening assay only; should not be directly extrapolated to the complex signaling and regulation of the intact endocrine system in vivo  In current form, does not include metabolic activation (but could be adapted)  Chemicals that may denature the receptor protein (surfactants or chemicals that can change the pH of the test system) may not be tested or can only be tested at low concentrations. |
| Strengths | - No animals - Quick - Could be high-throughput - Can provide some mechanistic information in WoE - Cell-free |
| Weaknesses/Limitations | - Only developed for human ERα - Only useful for screening purposes - No intrinsic metabolic capability - Screening assay only; should not be directly extrapolated to the complex signaling and regulation of the intact endocrine system in vivo - Does not distinguish between ERα agonists or antagonists |
| Opportunities | Useful for screening |
| Concentration Setting | The concentrations tested should remain within the solubility range of the test chemicals. |
| Test Acceptance criteria | - Reference substance behavior - Full concentration curves for reference controls (estrogen) should meet measures of performance for curve-fit parameters based on the FW and CERI protocols and historical control data - Sufficient range of concentration of test chemicals must be used to clearly define the top of the competitive binding curve - Variability at least concentration and among 3 independent runs should be reasonable and scientifically defensible - Data should be sufficient for quantitative assessment of ER binding |
| Animal Minimization | No animals used. |
| Animal Numbers | 0 |
| Solvent delivery | Dependent on the substances tested  Ethanol is the preferred solvent. Alternatively, if the highest concentration of the test chemical is not soluble in ethanol, DMSO may be used. The concentration of ethanol or DMSO, if used, in the final assay wells is 1.5% and may not exceed 2%. Prior to use, the solvent should be tested for interference with the assay’s performance |
| Species Effectiveness | Assay is for human ERα |
| Connection to existing regulatory frameworks | Listed in OECD 150  Mentioned in EFSA / ECHA guidance as it pertains to OECD GD150 |
| Commercial availability of the assays | List of proficiency substances are commercially available  Receptors can be purchased – need to report the supplier, catalog No., lot, species of receptor, active receptor concentration provided from supplier, certification from supplier |

**Androgen Receptor Binding (Rat Prostate Cytosol)**

| **Category** | **Description** |
| --- | --- |
| Test Guideline(s) | [OPPTS 890.1150](https://www.epa.gov/sites/default/files/2015-07/documents/final_890.1150_ar_bindng_assay_sep_10.5.11.pdf) |
| Adopted/published | October 2011 |
| Test species | Rat (Sprague-Dawley ventral prostate tissue homogenate) |
| Overview of assay | The assay measures the ability of a radiolabeled ligand [^3^H] (R1881) to interact with the androgen receptor (AR) in the presence of increasing concentrations of a test chemical. |
| Endpoint(s) | Binding affinity (IC_50_); relative binding affinity (RBA); estimate K_d_ and B_max_ (saturation) |
| Statistics | Saturation binding and competitive binding |
| Endocrine Pathway(s) addressed | Androgen |
| Validation status | Validated at the National Level based on Non-OECD in vitro screens (Conceptual Framework Level 2) Document |
| Domain(s) of applicability | Compounds that are soluble in a relevant solvent can be used. The test does not include a xenobiotic metabolizing system but could be used if the metabolite is known and could be isolated. |
| Strengths | - High-throughput - Can provide mechanistic information |
| Weaknesses/Limitations | - Results should not be directly extrapolated to the complex signaling and regulation of the intact endocrine system *in vivo* - Requires rat tissue - Only useful for screening purposes - This test guideline is intended to be used in conjunction with other guidelines in the OPPTS 890 series to make up the full screening battery under the EDSP |
| Opportunities | Useful for screening for androgen activity. |
| Concentration Setting | The concentrations tested should remain within the solubility range of the test chemicals |
| Test Acceptance criteria | It is strongly recommended that before running test chemicals, a lab demonstrate that it can meet the performance criteria for each of the standards (17β-estradiol and norethynodrel) in order to indicate that a technician is capable of performing the assay correctly and consistently.  Competitive bind:   - Ligand depletion is minimal. - The solvent control substance does not alter the sensitivity or reliability of the assay.   Saturation binding:   - Run consistency across 3 independent experiments. - Value of non-specific binding <50% of total binding at highest concentration. |
| Animal Minimization | Human AR is now available as a recombinant protein and could replace the use of rat prostate cytosol when successful validation has been completed. |
| Animal Numbers | Needs rat prostate tissue. |
| Solvent Delivery | Dependent on the substances tested. Solvent concentrations should be <3% for ethanol; <10% for DMSO) |
| Species Effectiveness | High conservation across species. |
| Connection to existing regulatory frameworks | Mentioned in OECD GD150, part of USEPA EDSP Tier 1 battery, mentioned in EFSA / ECHA guidance |
| Commercial availability | Contract Research Organizations can perform |

**Aromatase (Human Recombinant)**

| **Category** | **Description** |
| --- | --- |
| Test Guideline(s) | [OPPTS 890.1200](https://www.regulations.gov/document/EPA-HQ-OPPT-2009-0576-0004) |
| Adopted/published | October 2009 |
| Test species | Tissues from various species can be used – typically with human placenta or rat ovary. Cell lines expression aromatase recombinantly are used for the OPPTS guideline. |
| Description | Aromatase is the enzyme catalyzing the final step in synthesis of estrogens. Inhibition of aromatase can result in decreased levels of estrogens and impaired fecundity. A radioactive substrate (3H-androstenedione) and  NADPH are added to microsomes containing the aromatase (CYP19) and  reductase complex. ^3^H_2_O is released during the conversion of androstenedione  to estrone, and can be quantified as a direct measurement of aromatase activity  per unit reaction time. Competitive inhibition of aromatase activity by test  chemicals can be detected by serial reaction tubes containing increasing  concentrations of the chemical of interest. |
| Endpoint(s) | Formation of tritiated water (^3^H_2_O) as result of the conversion of androstenedione to estrone. |
| Statistical endpoint summary | IC_50_ calculation, 95% confidence intervals |
| Statistics | Curve is fitted by weighted least squares nonlinear regression analysis (concentrations expressed as log). |
| Endocrine Pathway(s) addressed | Estrogen |
| Validation status | Validated at the National Level based on Non-OECD in vitro screens (Conceptual Framework Level 2) Document |
| Domain(s) of applicability | Compounds that are soluble in a relevant solvent can be used. |
| Strengths | - High-throughput - Can provide mechanistic information - Can be animal free |
| Weaknesses/Limitations | - Since in cells, results should not be directly extrapolated to the complex signaling and regulation of the intact endocrine system *in vivo* - Sometimes uses animal tissue - Only useful for screening purposes - This test guideline is intended to be used in conjunction with other guidelines in the OPPTS 890 series to make up the full screening battery under the EDSP |
| Opportunities | Useful for screening for endocrine activity |
| Concentration Setting | A maximum concentration should be the limit of the solubility or 1 µM. It is recommended not to use a concentration lower than 0.001 µM for the highest concentration tested. The lowest concentration to be tested is generally 10^-10^ M, but lower concentrations may be tested. 8 concentrations per test substance should be tested. |
| Test Acceptance criteria | Prior to conducting the assay for evaluation of test chemicals, it is recommended that each technician conduct at least one single run of the positive control experiment to demonstrate assay proficiency. Evaluate these data against the following criteria:   - The suggested mean for aromatase activity in the absence of an inhibitor is at least 0.1 nmol/mg-protein/min. - The recommended mean background control activity is ≤ 10% of the full activity control. - The suggested coefficient of variation for replicates within each sample type and concentration of 4-OH ASDN is less than 15%. |
| Animal Minimization | No use of animals (if human tissue is used) |
| Animal Numbers | Donor animals for ovary tissues may be required. |
| Solvent delivery | Test chemicals should be prepared in buffer, ethanol, or DMSO. Selection of the solvent is based on the physical chemical properties of the test chemical. |
| Species Effectiveness | Depends on the species tested, but high conservation across species. |
| Connection to existing regulatory frameworks | Listed in “Revised Guidance Document 150 on Standardized Test Guidelines for Evaluating Chemicals for Endocrine Disruption. A Tier 1 EDSP test. |
| Commercial availability | Contract Research Organizations can perform. |

**Estrogen Receptor Binding Assay Using Rat Uterine Cytosol (ER-RUC)**

| **Category** | **Description** |
| --- | --- |
| Test Guideline(s) | [OPPTS 890.1250](https://www.regulations.gov/document/EPA-HQ-OPPT-2009-0576-0005) |
| Adopted/published | October 2009 |
| Test species | Rat (Sprague-Dawley uterine cytosol) |
| Overview of assay | The assay measures the ability of a radiolabeled ligand (17β-estradiol) to interact with the estrogen receptor (ER) in the presence of increasing concentrations of a test chemical. |
| Endpoint(s) | Binding affinity (IC_50_); relative binding affinity (RBA); estimate K_d_ and B_max_ (saturation) |
| Statistics | Saturation binding and competitive binding |
| Endocrine Pathway(s) addressed | Estrogen |
| Validation status | Validated as part of the Interagency Coordinating Committee for the Validation of Alternative Methods (ICCVAM) |
| Validation approach | Following assay optimization, the protocol was tested for transferability to laboratories and for reliability across laboratories. Due to wider than expected intralaboratory variability in the first interlaboratory study, the protocol was modified and a second interlaboratory validation study was undertaken. In the second validation study, one stock solution was made in solvent and this solution diluted sequentially with buffer. The study was conducted with three laboratories and 23 chemicals. |
| Strengths | - High-throughput - Can provide mechanistic information |
| Weaknesses/Limitations | - Results should not be directly extrapolated to the complex signaling and regulation of the intact endocrine system *in vivo* - Requires animals to isolate cytosol - Only useful for screening purposes - “Older” version of ER binding assay that includes a mix of ERα and ERβ (though predominant in rat uterine cytosol is ERα) - This test guideline is intended to be used in conjunction with other guidelines in the OPPTS 890 series to make up the full screening battery under the EDSP |
| Opportunities | Useful for screening for estrogen activity. |
| Concentration Setting | The concentrations tested should remain within the solubility range of the test chemicals. |
| Test Acceptance criteria | Saturation binding:   - Run consistency across 3 independent experiments. - Increasing concentrations of unlabeled 17β-estradiol displace [H]-17β-estradiol from the receptor in a manner consistent with one-site competitive binding. - Ligand depletion is minimal. - The solvent control substance does not alter the sensitivity or reliability of the assay. - The test chemical was tested over a concentration range that fully defines the top of the curve   Competitive binding:   - Value of non-specific binding <50% of total binding at highest concentration |
| Animal Minimization | This is an *in vitro* test (but it does use animal tissue) |
| Animal Numbers | Needs rat prostate tissue |
| Solvent delivery | Dependent on the substances tested. Solvent concentrations should be <3% for ethanol; <10% for DMSO) |
| Species Effectiveness | High conservation across species; specific for rat ER |
| Connection to existing regulatory frameworks | Listed in “Revised Guidance Document 150 on Standardized Test Guidelines for Evaluating Chemicals for Endocrine Disruption. A Tier 1 EDSP test and mentioned in EFSA / ECHA guidance |
| Commercial availability of the assays | Contract Research Organizations can perform |

**Estrogen Receptor Transcriptional Activation (Human Cell Line HeLa-9903) Assay**

| **Category** | **Description** |
| --- | --- |
| Test Guideline(s) | [OPPTS 890.1300](https://www.regulations.gov/document/EPA-HQ-OPPT-2009-0576-0006) |
| Adopted/published | October 2009 |
| Test species | human(h) ERα-HeLa-9903 cell line |
| Overview of assay | Estrogen receptor agonists can be determined by exposing the cells to test substances. Changes in luciferase compared to controls will show estrogenic receptor activation. |
| Endpoint(s) | Luciferase |
| Statistics | Both qualitative (e.g., positive/negative) and/or quantitative (e.g., EC_50_) assessments of ER-mediated activity should be based on empirical data and sound scientific judgment. Where possible, positive results should be characterized by both the magnitude of the effect as compared to the vehicle (solvent) control or reference estrogen and the concentration at which the effect occurs. |
| Endocrine Pathway(s) addressed | Estrogen |
| Validation status | The Interagency Coordinating Committee on the Validation of Alternative Methods (ICCVAM) validated a similar test, the LUMI-CELL® ER (BG1Luc ER TA) Test Method, An In Vitro Assay for Identifying Human Estrogen Receptor Agonist and Antagonist Activity of Chemicals (enclosed). Based on this evaluation, ICCVAM recommended that the accuracy and reliability of the BG1Luc ER TA test method support its use as a screening test to identify substances with *in vitro* ER agonist or antagonist activity and concludes that the accuracy of this assay is at least equivalent to US EPA’s OPPTS 890.1300: Estrogen Receptor Transcriptional Activation (Human Cell Line (HeLa- 9903)). |
| Domain(s) of applicability | Substances that are water soluble or in their correct solvent can be tested in this assay.  Special considerations will need to be applied to highly volatile compounds. In such cases, nearby control wells may generate false positives, and this should be considered in light of expected and historical control values. This test system cannot capture metabolic activation. |
| Strengths | - High-throughput - Can provide mechanistic information - Animal free |
| Weaknesses/Limitations | - Since in cells, results should not be directly extrapolated to the complex signaling and regulation of the intact endocrine system *in vivo* - Only useful for screening purposes - This test guideline is intended to be used in conjunction with other guidelines in the OPPTS 890 series to make up the full screening battery under the EDSP - Downstream interactions would not be measured by this assay - Some substances display both agonist and antagonist activity (could be cell type dependent). Chemicals that are negative could be evaluated in an ER binding assays before concluding the substance does not bind the receptor. - Potential for chemicals to increase chemiluminescence via non-ER mechanisms, thus possibly giving a false positive response. |
| Opportunities | Useful for screening for endocrine activity |
| Concentration Setting | The concentrations tested should remain within the solubility range of the test chemicals and not demonstrate cytotoxicity while covering several orders of magnitude. |
| Test Acceptance criteria | - Data should be sufficient for a quantitative assessment of ER activation (for agonist assay) (i.e., efficacy and potency). - The mean reporter activity for the reference concentration of reference estrogen should be at least four times that of the vehicle control on each plate to ensure adequate sensitivity. - The concentrations tested should remain within the solubility range of the test chemicals and not demonstrate cytotoxicity. - Consistent results should be achieved in at least two out of two or three runs of the assay. - Demonstration of laboratory proficiency with 10 proficiency chemicals is required at the outset. - The assay also requires a minimum of 80% cell viability. |
| Animal Minimization | No animals are used |
| Animal Numbers | 0 |
| Non-solvent delivery | Dependent on the substances tested. DMSO, water, and ethanol (95% to 100% purity) are suitable solvents. In case DMSO is used as solvent, the maximum concentration of DMSO during incubation should not exceed 1% (v/v). Prior to use, the solvent should be tested for absence of cytotoxicity and interference with the assay performance |
| Species Effectiveness | The cell line is human derived. Could miss agonists in other species because of differences in receptors sequence. |
| Connection to existing regulatory frameworks | Listed in “Revised Guidance Document 150 on Standardized Test Guidelines for Evaluating Chemicals for Endocrine Disruption. A Tier 1 EDSP test. |
| Commercial availability of the assays | Proficiency substances and luciferase kits are commercially available.  The stably transfected hERα-HeLa-9903 cell line should be used for the assay. The cell line can be obtained from the Japanese Collection of Research Bioresources (JCRB) Cell Bank2, upon signing a Material Transfer Agreement (MTA). |

**Steroidogenesis (Human Cell Line – H295R) Assay**

| **Category** | **Description** |
| --- | --- |
| Test Guideline(s) | [OCSPP 890.1550](https://www.epa.gov/sites/default/files/2015-07/documents/final_890.1550_steroidogenesis_assay_sep_8.1.11.pdf) |
| Adopted/published | July 2011 |
| Test species | Human (h) H295R cell line |
| Overview of assay | Inducers and inhibitors of the production of testosterone and 17β-estradiol are determined by exposing H295R cells to test substances. Changes in the production of each hormone are compared to the solvent controls and increases and decreases are reported as fold-changes relative to these controls. |
| Endpoint(s) | Concentrations of testosterone and 17b-estradiol as determined by validated enzyme-linked immunoassays (ELISA), radio-immuno assay (RIA) or LC-MS. |
| Statistics | Results should be normalized to the mean solvent control value of each test plate, and results expressed as changes relative to the control (solvent control) in each test plate. All data are to be expressed as mean ± 1 standard deviation (SD). Depending on the concentration-response profile, data should be evaluated either using type 1 (i.e., parametric or non-parametric ANOVA followed by the appropriate post-hoc tests [Dunnett’s Test or Mann Whitney U Test] for incomplete concentration response curves where the two greatest concentrations are on the linear portion of the curve) or type 2 (regression models where a full concentration-response curve can be modeled) statistics. |
| Endocrine Pathway(s) addressed | Steroidogenesis |
| Validation status | Validation studies were conducted and completed under the guidance by the OECD Non Animal Validation Management Group following an international ring-test format using 28 test compounds. During this validation study the relevance and reliability of the H295R Steroidogenesis Assay has been demonstrated for its intended purpose. |
| Validation approach | Using known endocrine active compounds with diverse mechanisms of action including strong, medium, and weak inducers of different steroidogenic processes as well as negative compounds. |
| Domain(s) of applicability | Substances that are water soluble or in their correct solvent can be tested in this assay. Special considerations will need to be applied to those compounds that are highly volatile. In such cases, nearby control wells may generate false positives, and this should be considered in light of expected and historical control values. In the few cases where volatility may be of concern, the use of “plate sealers” may help to effectively isolate individual wells during testing and is therefore recommended in such cases. This test system only captures very limited metabolic activation that is described incompletely for this cell line. |
| Strengths | - High-throughput - Can provide mechanistic information - Animal free - Integrative effect on hormone production capturing all processes along the steroidogenic pathway - Can be modified to target specific enzymes |
| Weaknesses/Limitations | - Since in cells, results should not be directly extrapolated to the complex signaling and regulation of the intact endocrine system *in vivo.* - Other mechanisms through which ED can occur, including (i) interactions with hormone receptors, (ii) metabolic activation and/or inactivation of hormones, (iii) distribution of hormones to target tissues, and (iv) clearance of hormones from the body. None of the test methods under this TG addresses these modes of action. - Some substances may interact with antibody-based (e.g. ELISA) hormone detection assays. All compounds to be tested have to be assessed for potential cross-reactivity. Does not apply to LC-MS hormone detection systems. - H295R cells change their hormone-producing properties as a function of age (freeze-thaw cycles and passages); therefore, cells have to be grown using a specific pattern and assessed for baseline performance prior to commencing an experiment. |
| Opportunities | Useful for screening for endocrine activity |
| Concentration Setting | The concentrations tested should remain within the solubility range of the test chemicals and not demonstrate cytotoxicity. Commonly, the assay is conducted in two tiers, with tier one typically covering a concentration range between 0.001 – 1000 mM if no a priori information on chemical solubility, cytotoxicity and other properties is available, and with tier 2 bracketing the active concentrations identified during tier 1 using half-log concentration spacing. |
| Test Acceptance criteria | Before using the assay, a laboratory should demonstrate that it is capable of achieving and maintaining appropriate cell culture and test conditions required for the successful conduct of the assay using a set of six chemicals. When analyzed with the hormone measurement assay, the average recovery rates (based on triplicate measures) for the spiked amounts of hormone should not deviate more than 30% from nominal concentrations. No substantial (≥30 % of basal hormone production of the respective hormone) cross-reactivity with any of the hormones produced by the cells should occur. Prior to initiating a new series of experiments, a quality control experiment is to be conducted with the current batch of cells using a known inducer (forskolin) and inhibitor (prochloraz) of T and E2 production. Studies must follow acceptable ranges and/or variation for H295R assay test plate parameters. A minimum of two independent runs of the assay should be conducted. If consistent results have been achieved, then no further replications are required. In cases were results were ambiguous in the first two runs a third experiment has to be conducted. The minimum cell viability required per well is 80% relative to the average viability in the controls and wells with lower viability should not be included in the final data analysis. |
| Animal Minimization | No animals are used. |
| Animal Numbers | 0 |
| Solvent delivery | DMSO (95% to 100% purity) has been the only solvent validated for use with the assay to date. In case DMSO is used as solvent, the maximum concentration during incubation should not exceed 1% (v/v). If other solvents are intended to be used, they should be tested for absence of cytotoxicity and interference with assay performance. |
| Species Effectiveness | The cell line is human derived. However, given the conservation of the steroidogenic pathway across vertebrates it is assumed that this assay is applicable to all vertebrate taxa including fish, amphibians, reptiles, birds, and mammals. |
| Connection to existing regulatory frameworks | Listed in “Revised Guidance Document 150 on Standardized Test Guidelines for Evaluating Chemicals for Endocrine Disruption and part of the Tier 1 EDSP |
| Commercial availability of the assays | H295R cells are available from ATCC (NCI-H295R). Hormone analytical kits are available from multiple manufacturers; however, they performance needs to be validated before use with the assay. |

**Human Sodium/Iodide Symporter (NIS) Inhibition Radioactive Iodide Uptake (RAIU) Assay**

| **Category** | **Description** |
| --- | --- |
| Assay technology/name | [Human Sodium/Iodide Symporter (NIS) inhibition radioactive iodide uptake (RAIU) assay](https://www.sciencedirect.com/science/article/pii/S0887233316302569?via%3Dihub) |
| Adopted/published | Original assay development: 2017 |
| Test species | Human cell line |
| Description | A cell line (hNIS-HEK293T-EPA) was generated with stable transgene expression of a human SLC5A5 (NIS) cDNA clone which is downstream of a GFP. Inhibitors of human sodium iodide symporter activity are determined by exposing the cell line to test chemicals along with uptake buffer containing ^125^I, incubating, then washing the cells and measuring intracellular ^125^I. A cell viability assay is conducted in parallel to identify instances where inhibition is related to cytotoxicity. |
| Endpoint(s) | ^125^I uptake by the cells which is indicative of NIS activity. |
| Statistical endpoint summary | Raw data, measured in counts per minute (CPM), were normalized per assay plate with the mean CPM of DMSO control wells and expressed as percent of control (% control). Normalized active chemical concentration responses were fit using a four-parameter logistic curve model with top and bottom asymptotes constrained to 100 & 0, respectively. Assay raw readings were measured as CPM and normalized per well plate as the percent activity of the median DMSO control CPM value. Dose-response curves were fitted using the Hill model provided in U.S. EPA’s ToxCast Pipeline R package, which fits curves based on three models: constant, constrained Hill model, and constrained gain-loss model. |
| Statistics | Assay raw readings were measured as counts per minute (CPM) and normalized per 96-well plate as the percent activity of the median DMSO control CPM value (n=12). Dose-response curves were fitted using the Hill model provided in U.S. EPA’s ToxCast Pipeline R package, which fits curves based on three models: constant, constrained Hill model, and constrained gain-loss model. |
| Endocrine Pathway(s) addressed | Thyroid |
| Validation status | Not validated |
| Domain(s) of applicability | Compounds that are soluble in a relevant solvent can be used. This test system cannot capture metabolic activation |
| Strengths | - No animals - MIE specific - Can provide mechanistic information - Amenable for high throughput screening 96-well format |
| Weaknesses/Limitations | - *In vitro* cell based assays do not inform on the feedback signaling and regulation of the intact thyroid pathway   This test is designed to detect inhibitors of a single molecular target (i.e., NIS) within the thyroid hormone pathway, and thus does not address other mechanisms through which thyroid disruption can occur including: (i) interference with other aspects of thyroid hormone synthesis; (ii) distribution of thyroid hormones to target tissues; (iii) transport of thyroid hormone into target cells; (iv) interactions with receptors and other enzymes within the thyroid hormone pathway; and (v) clearance of thyroid hormones from the body.   - Downstream interactions would not be measured by this assay |
| Opportunities | Could be used for screening and prioritization for thyroid activity |
| Concentration Setting | The concentrations tested should remain within the solubility range of the test chemicals in water or DMSO. High throughput screening is initially performed in single-concentration format (100uM) followed by further testing of active chemicals in multiple-concentrations (1nM -100uM). A concentration response including six test concentrations spanning a 10,000-fold range is recommended. |
| Test Acceptance criteria | - A Z’ factor value of >0.5 indicates good separation between the positive and negative controls and should be considered an acceptable plate run. - Consistent performance of the positive control (NaClO_4_) for potency and concentration-response curve. - The concentrations tested should remain within the solubility range of the test chemicals and not demonstrate cytotoxicity at all concentrations. - Consistent results should be achieved in at least two out of three runs of the assay. To be acceptable, the results should also meet the performance criteria set for the assay. Small deviations are unlikely to compromise the assay, but judgement should be made on a case-by-case basis. |
| Animal Minimization | No animals are used. |
| Animal Numbers | 0 |
| Non-solvent delivery | Assay is conducted using DMSO as the solvent and the maximum concentration of DMSO during incubation should not exceed 1% (v/v). Other solvents, such as water, acetone or ethanol (95% to 100% purity) could also be used. |
| Species Effectiveness | This method used a human HEK cell line stably transfected with human NIS. However, NIS is fairly conserved protein in structure among vertebrates, so the results could be applicable to other vertebrates. |
| Connection to existing regulatory frameworks | This assay has not been incorporated into any formal regulatory framework yet. |
| Commercial availability | All supplies to perform the assay are commercially available and the hNIS-HEK293T-EPA cell line is available from the U.S.EPA by request. |

**Human Iodothyronine Deiodinase (DIO1) inhibition assay, colorimetric iodide**

| **Category** | **Description** |
| --- | --- |
| Assay technology/name(s) | [Human Iodothyronine Deiodinase (DIO1) inhibition assay, colorimetric iodide](https://academic.oup.com/toxsci/article/162/2/570/4706013) |
| Adopted/published | Original assay development: 2018 |
| Test species | Human recombinant iodothyronine deiodinase type l |
| Description | Inhibitors of human iodothyronine deiodinase type I (DIO1) enzyme activity are determined by exposing test chemicals in a cell-free assay using enzyme generated in an adenovirus expression system. Inhibition is measured using non-radioactive determination of iodide release by the Sandell-Kolthoff method in a 96-well plate format. |
| Endpoint(s) | The rate of yellow-colored cerium IV to the non-colored cerium III by arsenic increase in the presence of iodide in a concentration-dependent manner to measure deiodinase-liberated iodide |
| Statistical endpoint summary | Data was processed by determining the change in absorbance between the 1 and 10 minute reading for each well. The net change in absorbance for each well was determined by subtracting the mean background change in absorbance defined by the completely inhibited reaction in the six wells in that plate containing 200 μM PTU. Finally, all data were normalized by converting to % control of the mean of seven uninhibited reactions representing the maximum DIO1 activity (net change in absorbance of the DMSO control wells). The median of the three replicates was calculated and test chemical results were reported as percent inhibition, which was calculated as 100% minus percent of control uninhibited reaction. |
| Statistics | For those chemicals tested in concentration-response mode, data were analyzed with the ToxCast Analysis Pipeline package which fits curves based on three models: constant, constrained Hill model, and constrained gain-loss model. |
| Endocrine Pathway(s) addressed | Thyroid |
| Validation status | Not validated |
| Domain(s) of applicability | Compounds that are soluble in a relevant solvent can be used. This test system cannot capture metabolic activation. Iodine, osmium, ruthenium, manganese (with bromides), nitrite, thiocyanate, iron, fluoride, silver, mercury, and cyanide have been shown to affect the reaction, but chemicals containing these elements cannot be ruled out as untestable. |
| Strengths | - No animals - MIE-specific - Can provide mechanistic information - Quick (exposures are 3 hours; measurement takes 10 minutes) - 96-well format |
| Weaknesses/Limitations | - Cell free assays do not inform on the complex signaling and regulation of the intact thyroid pathway - Assay is focused on a single molecular target (DIO1), and thus does not address other mechanisms by which thyroid disruption can occur (e.g., thyroid hormone synthesis, transport, clearance, and interaction with receptors and other enzymes). - Downstream interactions would not be measured by this assay - Potential for chemicals to increase free iodide in the reaction not involving deiodinase or serve as a substrate for DIO1, thus possibly giving false responses. - Protein denaturation of the deiodinase enzyme could possibly affect the assay. |
| Opportunities | Could be used for screening and prioritization for thyroid activity |
| Concentration Setting | The concentrations tested should remain within the solubility range of the test chemicals in DMSO. Screening was performed first in single-concentration and then with multiple concentrations. A dose response including seven test concentrations spanning a 10,000 fold range is recommended. |
| Test Acceptance criteria | - A Z’ factor value of >0.5 indicates good separation between the positive and negative controls and should be considered an acceptable plate run. - Consistent performance of the positive control (PTU) for potency and concentration-response curve. - A plate-wise DMSO median absolute deviation (DMSO-MAD) should be calculated to define the variability around the uninhibited enzyme activity in the DMSO wells in each plate and should be consistently low. - A plate-wise positive median absolute deviation (PTU-MAD) should be calculated to describe the variability around the fully inhibited assay response produced by the wells with positive control (PTU) and should be consistently low. - The concentrations tested should remain within the solubility range of the test chemicals and not demonstrate protein denaturation or chemical aggregation effects. - Consistent results should be achieved in at least two out of two or three runs of the assay. To be acceptable, the results should also meet the performance standards given in the assay. Small deviations are unlikely to compromise the assay, but judgement should be made on a case-by-case basis. |
| Animal Minimization | No animals are used. |
| Animal Numbers | 0 |
| Solvent delivery | Assay has only been run with DMSO as the solvent and the maximum concentration of DMSO during incubation should not exceed 1% (v/v). Other solvents, such as water, acetone or ethanol (95% to 100% purity) could be tested. |
| Species Effectiveness | The enzyme is human derived, however, DIO1 is a fairly conserved enzyme in structure, so there is the possibility that results could be applicable to other vertebrates. |
| Connection to existing regulatory frameworks | This assay has not been incorporated into any formal regulatory framework yet. |
| Commercial availability | All supplies to perform the assay are commercially available. (NOTE: Current assay used recombinant DIO1 produced in-house.) |

**Human Iodothyronine Deiodinase (DIO2) inhibition assay, colorimetric iodide**

| **Category** | **Description** |
| --- | --- |
| Assay technology/name(s) | [Human Iodothyronine Deiodinase (DIO2) inhibition assay, colorimetric iodide](https://academic.oup.com/toxsci/article/168/2/430/5250701) |
| Test Guideline(s) | N/A |
| Adopted/published | Original assay development: 2019 |
| Test species | Human recombinant iodothyronine deiodinase type 2 |
| Description | Inhibitors of human iodothyronine deiodinase type 2 (DIO2) enzyme activity are determined by exposing test chemicals in a cell-free assay using enzyme generated in an adenovirus expression system. Inhibition is measured using non-radioactive determination of iodide release by the Sandell-Kolthoff method in a 96-well plate format. |
| Endpoint(s) | The rate of yellow-colored cerium IV to the non-colored cerium III by arsenic increase in the presence of iodide in a concentration-dependent manner to measure deiodinase-liberated iodide |
| Endpoint Measurement and Summary | Data was processed by determining the change in absorbance between the 1 and 10 minute reading for each well. The net change in absorbance for each well was determined by subtracting the mean background change in absorbance defined by the completely inhibited reaction in the six wells in that plate containing 200 μM xanthohumol. Finally, all data were normalized by converting to % control of the mean of seven uninhibited reactions representing the maximum DIO2 activity (net change in absorbance of the DMSO control wells). The median of the three replicates was calculated and test chemical results were reported as percent inhibition, which was calculated as 100% minus percent of control uninhibited reaction. |
| Analysis/Statistics | For those chemicals tested in concentration-response mode, data were analyzed with the ToxCast Analysis Pipeline package version 1.0 using R version 3.3.1  which fits curves based on three models: constant, constrained Hill model, and constrained gain-loss model. |
| Endocrine Pathway(s) addressed | Thyroid |
| Validation status | Not validated |
| Domain(s) of applicability | Compounds that are soluble in a relevant solvent can be used. This test system cannot capture metabolic activation. Iodine, osmium, ruthenium, manganese (with bromides), nitrite, thiocyanate, iron, fluoride, silver, mercury, and cyanide have been shown to affect the reaction, but chemicals containing these elements cannot be ruled out as untestable. |
| Strengths | - No Animals - MIE-specific - Can provide mechanistic information - Quick (exposures are 3 hours; measurement takes 10 minutes) - 96-well format |
| Weaknesses/Limitations | - Cell free assays do not inform on the complex signaling and regulation of the intact thyroid pathway - Assay is focused on a single molecular target (DIO2), and thus does not address other mechanisms by which thyroid disruption can occur (e.g., thyroid hormone synthesis, transport, clearance, and interaction with receptors and other enzymes). - Downstream interactions would not be measured by this assay - Potential for chemicals to increase free iodide in the reaction not involving deiodinase or serve as a substrate for DIO2, thus possibly giving false responses. - Protein denaturation of the deiodinase enzyme could possibly affect the assay. |
| Opportunities | Could be used for screening and prioritization for thyroid activity |
| Concentration Setting | The concentrations tested should remain within the solubility range of the test chemicals in DMSO. Screening was performed first in single-concentration and then with multiple concentrations. A concentration response including seven test concentrations spanning a 10,000-fold range is recommended. |
| Test Acceptance criteria | - A Z’ factor value of >0.5 indicates good separation between the positive and negative controls and should be considered an acceptable plate run. - Consistent performance of the positive control (XTH) for potency and concentration-response curve. - A plate-wise DMSO median absolute deviation (DMSO-MAD) should be calculated to define the variability around the uninhibited enzyme activity in the DMSO wells in each plate and should be consistently low. - A plate-wise positive median absolute deviation (XTH-MAD) should be calculated to describe the variability around the fully inhibited assay response produced by the wells with positive control (XTH) and should be consistently low. - The concentrations tested should remain within the solubility range of the test chemicals and not demonstrate protein denaturation or chemical aggregation effects. - Consistent results should be achieved in at least two out of two or three runs of the assay. To be acceptable, the results should also meet the performance standards given in the assay. Small deviations are unlikely to compromise the assay, but judgement should be made on a case-by-case basis. |
| Animal Minimization | No animals are used. |
| Animal Numbers | 0 |
| Solvent delivery | Assay has only been run with DMSO as the solvent and the maximum concentration of DMSO during incubation should not exceed 1% (v/v). Other solvents, such as water, acetone or ethanol (95% to 100% purity) could be tested. |
| Species Effectiveness | The enzyme is human derived, however, DIO2 is a fairly conserved enzyme in structure, so there is the possibility that results could be applicable to other vertebrates. |
| Connection to existing regulatory frameworks | This assay has not been incorporated into any formal regulatory framework yet. |
| Commercial availability | All supplies to perform the assay are commercially available. (NOTE: Current assay used recombinant DIO2 produced in-house.) |

**Human Iodothyronine Deiodinase (DIO3) inhibition assay, colorimetric iodide**

| **Category** | **Description** |
| --- | --- |
| Assay technology/name(s) | [Human Iodothyronine Deiodinase (DIO3) inhibition assay, colorimetric iodide](https://academic.oup.com/toxsci/article/168/2/430/5250701) |
| Adopted/published | Original assay development: 2019 |
| Test species | Human recombinant iodothyronine deiodinase type 3 |
| Description | Inhibitors of human iodothyronine deiodinase type 3 (DIO3) enzyme activity are determined by exposing test chemicals in a cell-free assay using enzyme generated in an adenovirus expression system. Inhibition is measured using non-radioactive determination of iodide release by the Sandell-Kolthoff method in a 96-well plate format. |
| Endpoint(s) | The rate of yellow-colored cerium IV to the non-colored cerium III by arsenic increase in the presence of iodide in a concentration-dependent manner to measure deiodinase-liberated iodide |
| Statistical endpoint summary | Data was processed by determining the change in absorbance between the 1 and 10 min reading for each well. The net change in absorbance for each well was determined by subtracting the mean background change in absorbance defined by the completely inhibited reaction in the six wells in that plate containing 200 μM xanthohumol. Finally, all data were normalized by converting to % control of the mean of seven uninhibited reactions representing the maximum DIO3 activity (net change in absorbance of the DMSO control wells). The median of the three replicates was calculated and test chemical results were reported as percent inhibition, which was calculated as 100% minus percent of control uninhibited reaction. |
| Statistics | For those chemicals tested in concentration-response mode, data were analyzed with the ToxCast Analysis Pipeline package which fits curves based on three models: constant, constrained Hill model, and constrained gain-loss model. |
| Endocrine Pathway(s) addressed | Thyroid |
| Validation status | Not validated |
| Domain(s) of applicability | Compounds that are soluble in a relevant solvent can be used. This test system cannot capture metabolic activation. Iodine, osmium, ruthenium, manganese (with bromides), nitrite, thiocyanate, iron, fluoride, silver, mercury, and cyanide have been shown to affect the reaction, but chemicals containing these elements cannot be ruled out as untestable.  This test system cannot capture metabolic activation of test compounds. |
| Strengths | - No Animals - MIE-specific - Can provide mechanistic information - Quick (exposures are 3 hours; measurement takes 10 minutes) - 96-well format |
| Weaknesses/Limitations | - Cell free assays do not inform on the complex signaling and regulation of the intact thyroid pathway - Assay is focused on a single molecular target (DIO3), and thus does not address other mechanisms by which thyroid disruption can occur (e.g., thyroid hormone synthesis, transport, clearance, and interaction with receptors and other enzymes). - Downstream interactions would not be measured by this assay - Potential for chemicals to increase free iodide in the reaction not involving deiodinase or serve as a substrate for DIO3, thus possibly giving false responses. - Protein denaturation of the deiodinase enzyme could possibly affect the assay. |
| Opportunities | Could be used for screening and prioritization for thyroid activity |
| Concentration Setting | The concentrations tested should remain within the solubility range of the test chemicals in DMSO. Screening was performed first in single-concentration and then with multiple concentrations. A concentration response including seven test concentrations spanning a 10,000-fold range is recommended. |
| Test Acceptance criteria | - Data should be sufficient for a quantitative assessment of DIO3 inhibition. - The response of the randomly assigned DMSO and xanthohumol (XTH) high concentration wells are used to calculate a Z’ factor for each plate - A Z’ factor value of >0.5 indicates good separation between the positive and negative controls and should be considered an acceptable plate run. - Consistent performance of the positive control (XTH) for potency and concentration-response curve. - A plate-wise DMSO median absolute deviation (DMSO-MAD) should be calculated to define the variability around the uninhibited enzyme activity in the DMSO wells in each plate and should be consistently low. - A plate-wise positive median absolute deviation (XTH-MAD) should be calculated to describe the variability around the fully inhibited assay response produced by the wells with positive control (XTH) and should be consistently low. - The concentrations tested should remain within the solubility range of the test chemicals and not demonstrate protein denaturation or chemical aggregation effects. - Consistent results should be achieved in at least two out of two or three runs of the assay. To be acceptable, the results should also meet the performance standards given in the assay. Small deviations are unlikely to compromise the assay, but judgement should be made on a case-by-case basis. |
| Animal Minimization | No animals are used. |
| Animal Numbers | 0 |
| Solvent delivery | Assay has only been run with DMSO as the solvent and the maximum concentration of DMSO during incubation should not exceed 1% (v/v). Other solvents, such as water, acetone or ethanol (95% to 100% purity) could be tested. |
| Species Effectiveness | The enzyme is human derived, however, DIO3 is a fairly conserved enzyme in structure, so there is the possibility that results could be applicable to other vertebrates. To investigate species similarities/differences and applicability of the human DIO3 assay results to other vertebrates, an assay was recently developed for amphibian DIO3 inhibition. Enzyme was generated by transfecting the *Xldio3* gene into HEK293 cells via recombinant pcDNA3.1(+) plasmid. The assay then closely follows the human DIO3 assay. |
| Connection to existing regulatory frameworks | This assay has not been incorporated into any formal regulatory framework yet. |
| Commercial availability | All supplies to perform the assay are commercially available. (NOTE: Current assay used recombinant DIO3 produced in-house.) |

**Human Iodotyrosine Deiodinase (IYD) inhibition assay, colorimetric iodide**

| **Category** | **Description** |
| --- | --- |
| Assay technology/name(s) | [Human Iodotyrosine Deiodinase (IYD) inhibition assay, colorimetric iodide](https://www.ncbi.nlm.nih.gov/pmc/articles/PMC8130633/pdf/nihms-1682375.pdf) |
| Adopted/published | Original assay development: 2020 |
| Test species | Human recombinant iodotyrosine deiodinase (IYD) |
| Description | Inhibitors of human iodotyrosine deiodinase (IYD) enzyme activity are determined by exposing test chemicals in a cell-free assay using enzyme generated in a baculovirus expression system. Inhibition is measured using non-radioactive determination of iodide release by the Sandell-Kolthoff method in a 96-well plate format. |
| Endpoint(s) | The rate of yellow-colored cerium IV to the non-colored cerium III by arsenic increase in the presence of iodide in a concentration-dependent manner to measure deiodinase-liberated iodide. |
| Statistical endpoint summary | Data was processed by determining the change in absorbance between the 1 and 10 minute reading for each well. The net change in absorbance for each well was determined by subtracting the mean background change in absorbance defined by the completely inhibited reaction in the six wells in that plate containing 200 μM PTU. Finally, all data were normalized by converting to % control of the mean of seven uninhibited reactions representing the maximum DIO1 activity (net change in absorbance of the DMSO control wells). The median of the three replicates was calculated and test chemical results were reported as percent inhibition, which was calculated as 100% minus percent of control uninhibited reaction. |
| Statistics | For those chemicals tested in concentration-response mode, data were analyzed with the ToxCast Analysis Pipeline package which fits curves based on three models: constant, constrained Hill model, and constrained gain-loss model. |
| Endocrine Pathway(s) addressed | Thyroid |
| Validation status | Not validated |
| Domain(s) of applicability | Compounds that are soluble in a relevant solvent can be used. This test system cannot capture metabolic activation. Iodine, osmium, ruthenium, manganese (with bromides), nitrite, thiocyanate, iron, fluoride, silver, mercury, and cyanide have been shown to affect the reaction, but chemicals containing these elements cannot be ruled out as untestable. |
| Strengths | - No Animals - MIE-specific - Can provide mechanistic information in WoE - Quick (exposures are 3 hours; measurement takes 15 minutes) - 96-well format |
| Weaknesses/Limitations | - Cell free assays do not inform on the complex signaling and regulation of the intact thyroid pathway - Assay is focused on a single molecular target (IYD), and thus does not address other mechanisms by which thyroid disruption can occur (e.g., thyroid hormone synthesis, transport, clearance, and interaction with receptors and other enzymes). - Downstream interactions would not be measured by this assay - Potential for chemicals to increase free iodide in the reaction not involving deiodinase or serve as a substrate for IYD, thus possibly giving false responses. - Protein denaturation of the deiodinase enzyme could possibly affect the assay. |
| Opportunities | Could be used for prioritization or screening. |
| Concentration Setting | The concentrations tested should remain within the solubility range of the test chemicals in DMSO. Screening was performed first in single-concentration and then with multiple concentrations. A dose response including seven test concentrations spanning a 10,000-fold range is recommended. |
| Test Acceptance criteria | - A Z’ factor value of >0.5 indicates good separation between the positive and negative controls and should be considered an acceptable plate run. - Consistent performance of the positive control (MNT) for potency and concentration-response curve. - A plate-wise solvent median absolute deviation (Solvent-MAD) should be calculated to define the variability around the uninhibited enzyme activity in the solvent control wells in each plate and should be consistently low. - A plate-wise positive median absolute deviation (MNT-MAD) should be calculated to describe the variability around the fully inhibited assay response produced by the wells with positive control (MNT) and should be consistently low. - The concentrations tested should remain within the solubility range of the test chemicals and not demonstrate protein denaturation or chemical aggregation effects. - Consistent results should be achieved in at least two out of two or three runs of the assay. To be acceptable, the results should also meet the performance standards given in the assay. Small deviations are unlikely to compromise the assay, but judgement should be made on a case-by-case basis. |
| Animal Minimization | No animals are used. |
| Animal Numbers | 0 |
| Non-solvent delivery | Assay has only been run with DMSO as the solvent for the test chemicals and the maximum concentration of DMSO during incubation should not exceed 1% (v/v). Other solvents, such as water, acetone or ethanol (95% to 100% purity) could be tested. MNT positive control was delivered in 0.05M NaOH. |
| Species Effectiveness | The enzyme is human derived, however, IYD is a fairly conserved enzyme in structure, so there is the possibility that results could be applicable to other vertebrates. To investigate species similarities/differences and applicability of the human IYD assay results to other vertebrates, an assay is in development for amphibian IYD inhibition. This assay uses liver microsomes from *Xenopus laevis* tadpoles as the IYD enzyme source, and then closely follows the human IYD assay. |
| Connection to existing regulatory frameworks | This assay has not been incorporated into any formal regulatory framework yet. |
| Commercial availability | All supplies to perform the assay are commercially available. (NOTE: Current assay used recombinant IYD produced in-house.) |

**Amplex UltraRed Thyroperoxidase Inhibition Assay**

| **Category** | **Description** |
| --- | --- |
| **Assay technology** | [**Amplex UltraRed Thyroperoxidase Inhibition Assay**](https://academic.oup.com/toxsci/article/151/1/160/2461638) |
| Adopted/published | - Screening implementation: 2016 - Original assay development: 2014 |
| Test species | Rat thyroid microsomes or human cell line |
| Description | Amplex UltraRed (AUR) can be used for the detection of H_2_O_2_ released from biological samples in the presence of excess horseradish peroxidase; AUR is a fluorogenic substrate that is converted from AUR to Amplex UltroxRed by horseradish peroxidase in the presence of H_2_O_2_. The AUR substrate can be used to detect peroxidase activity (i.e., TPO activity) in the presence of excess H_2_O_2_ for the AUR-TPO assay. |
| Endpoint(s) | Thyroperoxidase (TPO) inhibition |
| Statistical endpoint summary | For the AUR TPO assay as implemented for ToxCast, the raw fluorescence unit data were normalized to the median of the vehicle control well values. |
| Statistics | The ToxCast Pipeline was used to analyze single concentration and multi-concentration response data, with multi-concentration response data curve-fit as well using Hill, gain-loss, and constant models. A threshold for a positive response in multi-concentration screening was 3 times the baseline median absolute deviation of the two lowest test concentrations on a plate-by-plate basis. |
| Endocrine Pathway(s) addressed | Thyroid |
| Validation status | JRC/ECCVAM is currently experimentally validating this assay system in a 96 well plate format. |
| Validation approach | Chemical training set developed by review of the literature; repeated across two different screening campaigns. Also compared to existing guaiacol peroxidation assay technology for 150 chemicals. |
| Domain(s) of applicability | Substances that are water soluble or in their correct solvent can be tested in this assay. Special considerations will need to be applied to those compounds that are highly volatile. In such cases, nearby control wells may generate false positives, and this should be considered in light of expected and historical control values. In the few cases where volatility may be of concern, the use of “plate sealers” may help to effectively isolate individual wells during testing and is therefore recommended in such cases. Reactive chemicals may appear to nonspecifically inhibit protein (look to nonspecific protein inhibition assay and cytotoxicity assay). Thyroid microsomes or expressed TPO will not provide metabolic capability. |
| Strengths | - High-throughput - Can provide mechanistic information - 96 or 384 well plate versions depending on implementation |
| Weaknesses/Limitations | - Cell free assays do not inform on the complex signaling and regulation of the intact thyroid pathway. - If using rat thyroid microsomes, a limitation is obtaining this tissue. - Nonspecific protein inhibition should be ruled out. Proteases, detergents, or generic protein inhibitors would give a positive result. - Assay may work on other peroxidases (e.g., LPO), so may not be specific to TPO if using a tissue other than thyroid. This can be avoided by using cell-line derived TPO as well. - If desired, an orthogonal confirmation using a guaiacol TPO inhibition assay could be performed (see Paul Friedman et al 2016 for implementation). |
| Opportunities | Useful for screening for thyroid activity |
| Concentration Setting | The concentrations tested should remain within the solubility range of the test chemicals in DMSO. |
| Test Acceptance criteria | - Consistent performance of methimazole (MMI) as a lead compound - Use of a ‘Z’ threshold as a determinant (e.g., greater than 0.5 for the plate). |
| Animal Minimization | Yes – depends on implementation and if using rat thyroid microsomes. Approximately 87 test wells can be run with a single rat thyroid (approximately 15–20mg of tissue). |
| Animal Numbers | Can use rat thyroid tissue. |
| Solvent delivery | DMSO solvent for ToxCast implementation. |
| Species Effectiveness | Conservation of TPO across species (rat, porcine, human) |
| Connection to existing regulatory frameworks | - Level 2 in Table 9 of EFSA Guidance on identification of EDs - JRC/ECCVAM working to “validate” the AUR-TPO assay |
| Commercial availability | All supplies to perform the assay are commercially available, except for rat thyroid microsomes which are difficult to purchase. Human recombinant TPO may be purchased or generated. |
